# The Role of Fluorinated IL as an Interfacial Agent in P(VDF-CTFE)/Graphene Composite Films

**DOI:** 10.3390/nano9081181

**Published:** 2019-08-19

**Authors:** Jing Yang, Sébastien Pruvost, Sébastien Livi, Jannick Duchet-Rumeau

**Affiliations:** Laboratory of polymer materials Engineering, UMR CNRS 5223, INSA Lyon, University of Lyon, 69621 Villeurbanne, France

**Keywords:** graphene, fluorinated polymers, composites, interfacial effect

## Abstract

The incorporation of graphene into a polymer matrix can endow composites with extended functions. However, it is difficult to well disperse pristine graphene into a polymer matrix in order to obtain polymer nanocomposites due to the lack of functional groups on the surface for bonding with a polymer matrix. Herein, we investigated the role of fluorinated ionic liquid (IL) as a new interfacial agent in poly(vinylidene fluoride-co-chlorotrifluoroethylene) (P(VDF-CTFE))/graphene composite films. First, a task-specific IL, perfluorooctyltriphenylphosphonium iodide (IL-C8F13), was synthesized and adsorbed on the surface of graphene oxide (GO) and reduced graphene oxide (rGO) for making functional nanofillers which were capable of being incorporated into the P(VDF-CTFE) matrix. The cation structure of IL combined three phenyls (potential π–π interactions with graphene) and a short fluorinated chain (enhanced miscibility with fluorinated matrix via dipolar interactions) to make a compatible graphene filler and P(VDF-CTFE) matrix at the interface among them. Second, two series of P(VDF-CTFE)/GO-IL and P(VDF-CTFE)/rGO-IL composites with different loading contents were prepared with the goal of providing an understanding of the mechanism of interfacial interactions. This paper investigated the difference in the interaction model between GO with IL and rGO with IL. Subsequently, the interfacial effect of IL on the properties of P(VDF-CTFE)/graphene composites, such as crystallization, chain segmental relaxation behavior, dispersion, and the final dielectric properties will be further studied.

## 1. Introduction

It is well-known that the incorporation of nanofillers into a polymer matrix can endow the composites extended functions and applications originated from the functional fillers while retaining excellent processing and manufacturing abilities due to the flexibility inherent in polymer matrices [1]. Graphene, a monolayer of sp^2^ hybridized carbon atoms arranged in a hexagonal lattice, has potential applications for developing nanocomposites, sensors, supercapacitors, and optoelectronic devices, etc. due to its intriguing properties such as excellent electrical conductivity, ultrahigh aspect ratio and relatively low production cost [2,3]. Therefore, a great deal of research has been under the spotlight to prepare graphene/polymer nanocomposites [4]. Through the synergistical effect from both graphene and polymer, these nanocomposites could exhibit outstanding structural performances and multifunctional properties if the well-controlled structuration and interfacial organization can be achieved. However, it can be difficult to directly use pristine graphene as the nanofiller for polymer composites due to the lack of functional groups on the surface for bonding with polymer matrix. It is difficult to prevent the macroscopic aggregation of graphene sheets to restore the graphite-like one due to the inherent affinity between graphene layers via π–π stacking. Johnson et al. [5] detailed different ways to well disperse graphene in a review. Therefore, the fine control of the dispersion and distribution of graphene nanofillers in the polymer matrix and the interface tuning is of the utmost necessity and significance in order to obtain an enhanced performance of graphene/polymer nanocomposites [6].

Fluorinated polymers (poly(vinylidene fluoride) (PVDF) or its copolymers) are well known as ferroelectric materials and are polymers with a high dielectric constant which can make them relevant for energy storage or for electroactive materials. We focused on the preparation of poly(vinylidene fluoride-co-chlorotrifluoroethylene) (P(VDF-CTFE))/graphene nanocomposites and expected to obtain enhanced properties thanks to the combining of the merits of both graphene nanofillers and the electroactive PVDF-based matrix. For polymers with low electrical conduction, actuators applications can be considered, and, for higher electrical conductivity, composites can be used as material for electrodes. However, the naturally poor compatibility between non-polar graphene and a polar PVDF-based matrix is a negative factor in forming a homogeneous distribution, which gives rise to adverse excessive aggregation of graphene and vacancies at interfaces. In contrast, surface functional oxidized graphene (GO) with heavily oxygenated groups such as hydroxyl, epoxide, carbonyl, and carboxyl allow more compatible and homogeneous dispersion in the more, or rather, polar polymer matrix. However, GO is usually incompatible with most organic solvents which are good solvents for the polymer matrix. Moreover, unlike graphene, GO is considered electrically insulator, which is undesired to be used as the conductive filler in the composites [7]. Jung et al. [8] studied methods of tuning the electrical conductivity of graphene oxide sheets by reducing the number of sheets. They demonstrated that the electrical behavior of graphene oxide depends, in particular, on the number of defects and oxygenated groups on its surface. Composites with GO filler can be viewed as good candidates for actuator applications and/or for energy storage in the case of low electrical conductivity. A routine way to obtain conductive graphene filler in the matrix is the chemical reduction of GO to produce reduced oxidized graphene (rGO), recovering the graphitic sp^2^ network, but the aggregation issue should also be avoided during the reduction process [7] by adding an agent to facilitate the dispersion of very small fillers. The obtained composites can exhibit high dielectric constants if conductive fillers are separated from each other or can be used as electrodes if high electrical conductivity is achieved. More generally, the chemical natures of these different fillers (i.e., graphite, GO, and rGO) have an impact on their dispersion in the fluorinated matrix.

Therefore, the key strategy to enhancing the dispersion of graphene in the PVDF-based matrix is the surface functionalization, endowing the so-called interfacial agent on the surface of the graphene to tune the interfacial bonding between graphene and polymer matrix with controlled and reinforced properties. Several works are described in the literature to show the well-dispersed GO or rGO in PVDF-based matrix by either covalent or non-covalent surface modification of graphene. Poly(methyl methacrylate) (PMMA) chains have been grafted from the GO or rGO surface via surface-initiated atom transfer radical polymerization (SI-ATRP) [9]. PMMA covalently modified graphene layers become thinner for the exfoliation during composite formation promoting the formation of piezoelectric β-crystal and enhancing the thermal and mechanical properties of PVDF for only 5% of loading content. Poly(vinyl alcohol) (PVA) was used to be covalently bonded to the surface of GO through esterification reaction [10]. Interactions between PVDF and rGO-PVA were enhanced through the intermolecular hydrogen bonds between PVA and PVDF. Han et al. [11] reported the tetraethyl orthosilicate-modified rGO nanosheets as fillers incorporated in P(VDF-CTFE) matrix. The presence of SiO_2_ layers broke the connectivity of the fillers and reduced the ohmic conduction. Thus, the dielectric loss of the P(VDF-CTFE) composites containing r-GO@SiO_2_ was significantly reduced compared to that of r-GO-based ones.

More recently, ionic liquids (ILs) have also been used as modifiers of the surface of graphene [12]. Ionic liquids showed an efficient interfacial reinforcement between the PVDF matrix and GO fillers, resulting in nearly pure γ-crystals due to the introduction of strong ion–dipole interactions between PVDF and GO. A phosphonium IL, (1-hexadecyl)triphenylphosphonium bromide (HTPB), was used as an interfacial agent to tune the interface property [13]. The modified graphene, even after the reduction from GO (HTPB-rGO), still retained an excellent dispersion in the co-solvent of PVDF, i.e., dimethylacetamide (DMAc), without any macroscopic aggregation. Thus, the resulting HTPB-rGO/PVDF composite films exhibited a dielectric constant at 1 kHz, three times more than neat PVDF, one at a very low percolation threshold (0.662 wt%). Moreover, the interfacial agent HTPB can also efficiently induce polar β- or γ-phase through the interfacial interactions with the matrix to influence the chain segmental configuration of PVDF. Imidazolium ILs with a functional amino group were covalently bonded on the surface of GO to fabricate the PVDF/GO-IL composites through amidation reaction between –COOH on the edge of surface of GO and –NH_2_ of IL [14]. Due to the interfacial interactions between PVDF chains and GO-IL, a dendritic morphology, proof of a homogeneous dispersion in the PVDF matrix, was generated leading to an enhanced conductivity of 10^−2^ S/cm even at a low content (0.1 wt%) of GO-IL incorporated.

**Scheme 1:**
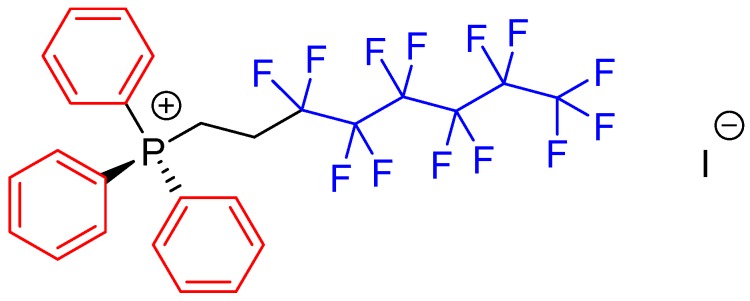
Chemical structure of perfluorooctyltriphenylphosphonium iodide (IL-C8F13).

Although a few works above have demonstrated that ILs used as interfacial agents can dramatically improve the dispersion of GO in PVDF matrix, some issues of the role of ILs as interfacial agents in polymer composites still need to be paid more attention, as follows. First, for most reported ILs used as a modifier, the commercial origin of ILs is short of chemical structure design. They only focus on the use of monofunctional cations with imidazolium or phenyls structures which may form π–π interactions with graphene. However, an ideal interfacial compatibilizer is preferred to have difunctional moieties, binding both graphene and polymer matrix to realize the interfacial reinforcement. In view of this consideration, a task-specific IL, perfluorooctyltriphenylphosphonium iodide (IL-C8F13) [15] (Scheme 1), will be synthesized and adsorbed on the surface of GO (GO-IL) and rGO (rGO-IL) as functional nanofillers incorporated into the P(VDF-CTFE) matrix.

The cation structure combines three phenyls suitable for potential π–π interaction with graphene and a short fluorinated chain to promote miscibility with fluorinated matrix via dipolar interaction [16]. Second, the usual strategy starting from GO to IL-modified GO and finally to reduced rGO-IL paid less attention to the change in the functional environment between graphene and IL during the reduction process, which is a key factor for influencing the interfacial interactions [17]. Thus, the difference in the interaction model of GO with IL and rGO with IL was investigated in this work. Third, the interfacial effect of IL was investigated on the dispersion achieved and the properties of P(VDF-CTFE) composites, such as crystallinity and the final dielectric properties of composites.

## 2. Materials and Methods

### 2.1. Materials

Graphite powder with a size ≤20 μm was purchased from Aldrich (St. Louis, MO, USA). Fluorinated phosphonium IL (IL-C8F13) was synthesized following a report described elsewhere [12]. In a typical synthesis, triphenylphosphine (20 mmol, 5 g) and 1*H*,1*H*,2*H*,2*H*-perfluorooctyl iodide (9.48 g, 20 mmol) were mixed in toluene (20 mL) at room temperature (RT). The stirred suspension was heated under reflux for 24 h at 120 °C and a white precipitate was formed. After cooling to room temperature, the reaction mixture was filtered then washed repeatedly with petroleum ether and dried under vacuum to produce white powder (yield = 65%). The H_2_SO_4_, NaNO_3_, and KMnO_4_ were purchased from CARLO ERBA (Paris, France) and used as received. The P(VDF-CTFE) copolymer containing 8 wt% of chlorotrifluoroethylene (CTFE) was purchased from Arkema (Colombes, France). The solvent dimethylformamide (DMF) was provided by CARLO ERBA (Paris, France) and used as received.

### 2.2. Preparation of GO and rGO

Graphene oxide (GO) was prepared by a modified Hummers method [18]. In a typical synthesis, graphite powder (1 g) and NaNO_3_ (0.75 g) were charged in a flask and concentrated H_2_SO_4_ (96%, 34 mL) was added into it at 0 °C. The mixture was allowed to stir overnight at RT. KMnO_4_ (5 g) was slowly added into the flask in 3 h at 0 °C, and then stirred at 0 °C for 2 h until the color became dark green. The flask was shifted to an oil bath and the mixture was allowed to stir at 38 °C for 3 days until it became pasty brownish. The temperature was slowly increased to 60 °C and allowed to stir for 4 h. The mixture was diluted with de-ionized water (40 mL) at 0 °C, and the reaction was ended by adding 7 mL of H_2_O_2_ (30%) solution at 0 °C until the color became dark yellow. The mixture was centrifuged for 10 min and the solid was washed by HCl solution (5%). After 10 cycles of centrifugation/washing with HCl solution, the solid was then washed with pure water 10 times. The resulting solid was suspended in water under ultrasonication for at least 12 h to exfoliate graphite oxide until no visible particle was observable by native eyes. The brown solution was then centrifuged for 10 min to remove a very small number of visible particles. The final GO colloid in water was lyophilized to obtain fluey GO solid. The GO product was then resuspended in water (1 mg/mL), after ultrasonication for 2 h, and the GO colloid in water with perfect dispersion was reobtained.

For the preparation of rGO, a solution of GO in DMF (0.5 g/L) was prepared by ultrasonic treatment for 1 h. The solution was then reduced by hydrazine hydrate H_2_N–NH_2_ (4 times in mass) at 95 °C for 3 h. The precipitates can be obtained during the process. After washing with water several times to remove the excess hydrazine and DMF, the product rGO was dried by lyophilization.

### 2.3. Modification of GO and rGO with IL: GO-IL and rGO-IL

In a typical synthesis, 0.5 mg/mL of GO colloid in water (4 mL) was prepared by ultrasonication for 20 min. The solution was alkalized by adding NaOH solution (0.1 M) till pH = 10. Two mL of IL-C8F13 solution in DMF (5 mg/mL) was then added dropwise under magnetic stirring (mass ratio of IL/GO = 5/1). The precipitant was then immediately observable. The solid was then separated by centrifugation and washed with water/DMF (2/1, v/v) 3 times and then water twice to remove the excess IL and DMF. The final product, GO-IL, was obtained by freeze-drying.

To prepare rGO-IL, a solution of GO-IL in DMF (0.5 g/L) was prepared by ultrasonic treatment for 1 h. The solution was then reduced by hydrazine hydrate (4 times in mass) at 95 °C for 3 h. The homogenous black suspension was concentrated and then water was added into the flask to obtain precipitates. After washing with water/DMF (2/1, v/v) and then water for several times to remove the excess hydrazine and DMF, the product rGO-IL was dried by lyophilization.

### 2.4. Preparation of P(VDF-CTFE)/GO, P(VDF-CTFE)/GO-IL, and P(VDF-CTFE)/rGO-IL Composite Films

A desired amount of GO, GO-IL or rGO-IL was ultrasonically dispersed in DMF for 1 h, and then P(VDF-CTFE) was added into the suspension. After magnetic stirring for 1 h, and ultrasonication for 2 h, the thin films were casted on a glass plate by doctor blade. The resulting composite films were firstly dried at 60 °C in an air oven overnight, and then under vacuum at 80 °C for 48 h to remove the residual solvent (no residual DMF was shown in the ^1^H NMR spectra of the final films, see Figure S1 in Supplementary Materials). Composite films, denoted as P(VDF-CTFE)/GO, P(VDF-CTFE)/GO-IL, and P(VDF-CTFE)/rGO-IL, with different compositions were prepared (see Table 1). According to the literature, the percolation threshold of 0.662 wt% was observed for phosphonium IL modified-rGO/PVDF composites [13], so the weight percentages of IL-modified graphene incorporated within a polymer matrix were finely adjusted from 0.27 to 2.70 wt% in this work. In order to investigate the effect of IL-C8F13 on the copolymer alone, the composites containing 0.5, 1, and 2 wt% of IL-C8F13 were also prepared.

### 2.5. Characterization

The ^1^H, ^13^C, ^19^F, and ^31^P NMR spectra were recorded in deuterated chloroform CDCl_3_ on a 400 MHz Bruker apparatus. The NMR spectra were used to control the stability of IL after reduction of GO by hydrazine.

The X-ray diffraction (XRD) data were collected using a Bruker D8 Advance X-ray diffractometer. A bent quartz monochromator (before the sample) was used to select the Cu Kα_1_ radiation (λ = 0.15406 nm) and was run under operating conditions of 45 mA and 33 kV in a Bragg–Brentano geometry. The angle range scanned was 1° < 2θ < 30° for film composites and 1° < 2θ < 50° for graphite, GO, and rGO powders at a scanning speed of 2°/min with a step interval of 0.02°. X-ray diffraction allows describing the graphene layers stacking in different fillers (graphite, GO, and rGO with or without IL) and the identification of crystalline phases of PVDF in all the composites.

Fourier transform infrared (FTIR) spectra were recorded at room temperature on a Nicolet iSO10 Thermo Scientific spectrometer. The attenuated total reflectance (ATR) modes (a diamond crystal was used) were performed to determine each characteristic band. All spectra were recorded at 2 cm^−1^ resolution and 32 scans from 4000 to 650 cm^−1^ for ATR mode. FTIR spectra help to characterize the functionalization of graphite after different treatments, the interactions established between filler, IL, and the polymer matrix and to identify the crystalline phases of PVDF in all the composites.

Thermogravimetric analysis (TGA) was performed on a Q500 thermogravimetric analyzer (TA instruments). The samples were heated from RT to 1000 °C at 10 K min^−1^ under nitrogen flow. Raman spectra were recorded on a DXR 532 nm filter system spectrometer (Thermo Fisher Scientific, Waltham, MA, USA) equipped with an integral microscope and a He–Ne laser (532 nm) was used as the excitation source.

Differential scanning calorimetry (DSC) analyses were conducted on a thermoanalyzer system calibrated with an Indium standard sample before each measurement, model Q20 (TA Co. Ltd., New Castle, DE, USA), under nitrogen atmosphere at a rate of 10 °C/min. Four scans (heating/cooling/heating/cooling, separated by a 10 min isotherm) were realized for each sample from −80 to 180 °C. All non-isothermal crystallization temperatures (*T*_c_) from the first cooling run and melting temperatures (*T*_m_) from the first and second heating run reported here correspond to the summit point of each peak.

Transmission electron microscopy (TEM) was performed at the Center of Microstructures (University of Lyon) using a Philips CM 120 field emission scanning electron microscope (Waltham, MA, USA) with an accelerating voltage of 80 kV. The samples were cut using an ultramicrotome equipped with a diamond knife to obtain 60 nm thick ultrathin sections. The stained sections by RuO_4_ were set on copper grids for observation. Scanning electron microscopy (SEM) was used to observe fractured cross-section morphologies of composite materials. The samples were fractured in liquid nitrogen and thin gold layers were deposited on the fractured surfaces of the samples prior to observations.

Dynamic measurements (mechanical and electrical) were carried out to study relaxations of the fluorinated matrix and to evaluate the impact of the different fillers on the amorphous phase. Dielectric analysis was also used to measure the dielectric constant and dielectric losses, essential parameters for energy applications. Dynamic mechanical analysis (DMA) was investigated by a Rheometrics Solid Analyzer RSA II (New Castle, DE, USA) at a frequency of 10 Hz. The heating rate was 3 K/min for the temperature range from −125 to 120 °C under a nitrogen atmosphere. Dielectric analysis (DEA) measurements were performed using a Novocontrol Alpha-A analyzer (BDS Novocontrol, Montabaur, Germany). Circular Au electrodes with a diameter of 1 cm were sputtered on thin films 2 × 2 cm^2^ with the thickness of 30–60 μm for the measurements. Measuring was done under the frequency of 10^7^~1 Hz and applying *V*_RMS_ = 1 V in the temperature range −80 to 120 °C with a heating rate of 3 K/min. The model used for measurement was metal–insulator–metal capacitor.

The direct-current (DC) electrical conductivities of composites with GO or IL at different temperatures were measured with a Keithley 2636 electrometer; at least two parallel measurements for each composition were conducted to verify the reproducibility. The temperature of the sample was controlled by an air-circulation oven from RT to 100 °C, and a thermometer was used to monitor the temperature of the air-circulation oven. The sample preparation was same as that used in DEA experiments.

## 3. Results

### 3.1. Characterizations of the Fillers

#### 3.1.1. Preparation of GO, rGO, GO-IL, and rGO-IL

**Figure 1:**
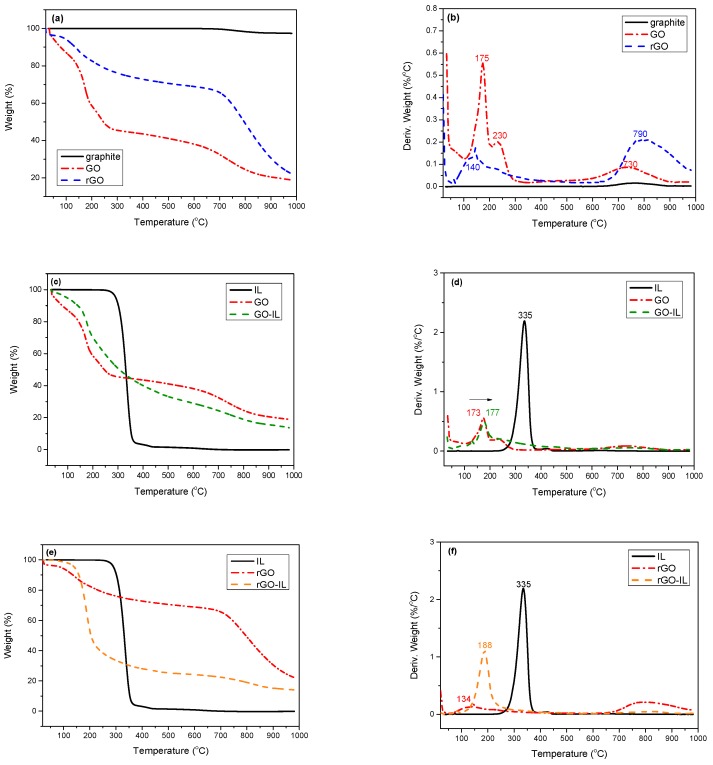
Thermogravimetric Analysis (TGA) (**a**) and derivative thermogravimetry (DTG) (**b**) thermograms of graphite, graphene oxide (GO), and reductive graphene oxide (rGO); TGA (**c**) and DTG (**d**) curves of GO, IL, GO-IL; TGA (**e**) and DTG (**f**) curves of rGO, IL, rGO-IL under N_2_ atmosphere.

Thermal stability of the fillers and interactions with IL were studied using thermogravimetric analysis. Figure 1a presents the thermogravimetric (TG) plots of graphite, GO, and rGO together with the derivatives of the TG signal, i.e., the so-called differential thermogravimetry (DTG) plots (Figure 1b). However, for GO, it was easy to observe a gradual mass loss in the range from RT to 100 °C. One possibility corresponded to the gradual removal of absorbed water because of the hydrophilic oxygen-containing functionalities covering the surface of the GO. Another possibility is due to the degradation of carboxylic acid on the edge of the graphene plane. The maximum weight loss which took place around 175 °C in the DTG curve of the GO was attributed to the decomposition of labile oxygen functional groups, and another relatively slow and steady mass loss beginning around 200 until 300 °C can be assigned to the elimination of more stable oxygen functionalities [19]. Moreover, the weight loss starting from 600 °C was ascribed to the carbon backbone sublimation [20,21]. After chemical reduction, the thermal stability of rGO improved compared to GO as represented in two aspects: (1) rGO did not show a gradual mass loss from RT to 100 °C because of the hydrophobic surface of rGO after reduction, and the peaks corresponding to the weight loss of oxygen functional groups became broader compared with those of GO due to the elimination of oxygenated groups by chemical reduction; (2) the carbon backbone decomposition of rGO initiated at approximately 700 °C which showed an approximately 100 °C of retardance compared to GO. It is important to note that IL-C8F13 is chemically stable under the reduction conditions by hydrazine (95 °C for 3 h in DMF with the same concentration), as confirmed by the identical NMR spectra before and after hydrazine treatment (see the ^1^H, ^13^C, ^19^F, and ^31^P NMR spectra in Figure S2). We cannot see the degradation peaks around 335 °C corresponding to ILs either from the DTG curves of GO-IL, shown in Figure 1d, or from rGO-IL, shown in Figure 1f. One possible explanation is that the amount of ILs attached onto the graphene surface was so limited that the observations of their degradation peaks are difficult. Another possibility is that the degradation corresponding to IL was associated with the degradation assigned to GO and rGO in the temperature range from 100 to 400 °C. However, we observed a slightly promoted influence of ILs on the thermostability of GO and rGO. As shown in the TGA and DTG curves in Figure 1c,e, IL only showed a rapid weight loss process from 250 to 400 °C, with a maximum weight loss at 335 °C. Moreover, it is interesting to see the higher temperature shifting of the maximum weight loss in the DTG curves of GO-IL and rGO-IL compared with GO and rGO, respectively. Firstly, this retardant effect of IL on the degradations of GO and rGO may reflect the interactions existing between IL and GO or rGO sheets. Secondly, the fluorinated IL, to some extent, improves thermostabilities of GO-IL and rGO-IL due to the relatively thermally stable nature of phosphonium IL with fluorinated chains.

**Figure 2:**
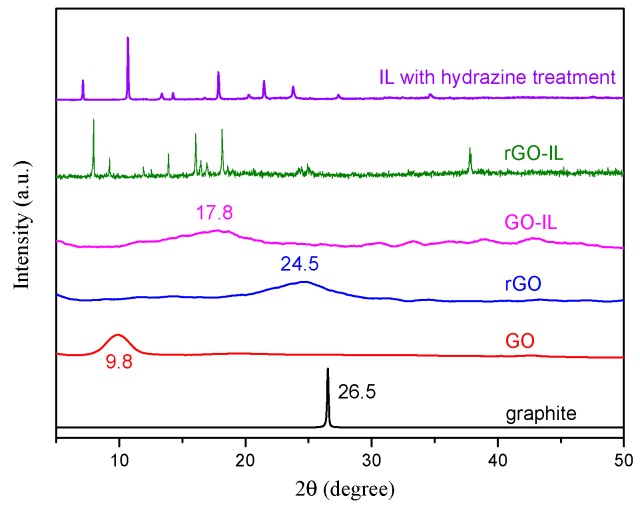
X-ray diffraction (XRD) patterns of graphite, GO, rGO, GO-IL, rGO-IL, and IL with hydrazine treatment.

The XRD patterns can be used to verify the preparation of GO, rGO, GO-IL, and rGO-IL. As shown in Figure 2, the pattern of graphite displays a (002) peak at 2θ = 26.5° characterizing the π-stacking interlayer distance (*d*) of 3.4 Å, which is very similar to the ones reported (*d* = 3.4 Å) in the literature [10,14,17,22,23]. Moreover, the average numbers of layers was calculated by the classic Debye–Scherrer equation:

$$t=\frac{0.89\lambda }{\beta \cos\theta }$$

$$n=\frac{t}{d}$$

where *t* = the thickness of the ordered stack of graphene sheets; λ = 1.5406 Å; β = full width at half maximum (FWHM) in radian; *n* = number of graphene layers; *d* = interlayer spacing [24,25]. Around 158 layers of graphene sheets were assembled for graphite, which indicates a multi-layered stacking structure for pristine graphite (all parameters from XRD patterns are summarized in Table 2).

After oxidation-induced expansion, the pattern of GO exhibited a (002) peak at 9.8° corresponding to an interlayer spacing of 9.0 Å, which is in good agreement with the ones reported in the literature (*d* = 9.1 Å) [14,17], because oxygenated functional groups generated on the GO surface increase the interlayer distance. The numbers of graphic layers decreased dramatically, as low as around four layers for GO, indicating a well exfoliated few-layer graphene structure. Nevertheless, the emergence of a new broad (002) peak at 24.5° in the XRD pattern of rGO indicates a reduced interlayer spacing of 3.6 Å (very similar to the ones reported, *d* = 3.6 Å [10,22]) due to the elimination of most oxygen-containing functional groups. After reduction, rGO still exhibits an exfoliated few-layer state (around four graphic layers), which is also significantly different from the pristine graphite [22]. As shown in Figure 2, GO-IL has a weak and broad (002) peak at 17.8° with a decreased interlayer spacing of 5.0 Å in comparison with GO, and its decreased interlayer spacing could be ascribed to the ILs absorbed on the surface of GO. The graphic layers calculated for GO-IL further decreased to around three, suggesting the positive influence of IL on the exfoliation. It is obvious to see a significantly different XRD pattern for rGO-IL: the broad diffraction of (002) peak of rGO at 24.5° disappeared but only some sharp peaks were observed. These sharp peaks could be partially due to the ILs modified on the rGO. Note that the ILs in the XRD experiments were conducted with the same hydrazine reduction treatment (95 °C for 3 h in DMF) used for the rGO. Moreover, the topology of exfoliated rGO-IL could have become highly disordered during non-covalent functionalization with IL, thus, the typical XRD diffraction pattern of rGO cannot be observed anymore for rGO-IL [17].

**Figure 3:**
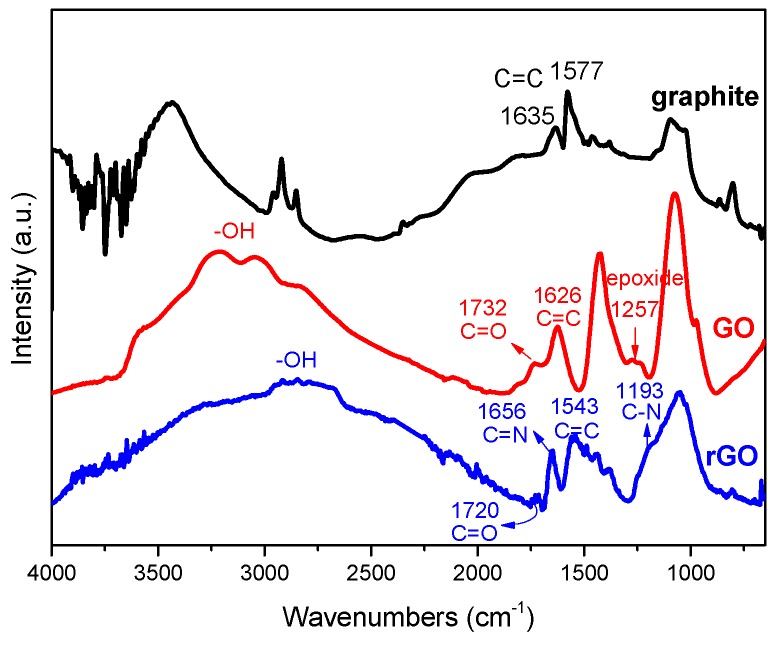
Attenuated total reflectance Fourier transform infrared (ATR-FTIR) spectra of graphite, GO, and rGO.

The IR spectrum of pristine graphite, shown in Figure 3, had two vibration peaks at 1635 and 1577 cm^−1^ ascribed to the C=C skeleton vibrations [26]. After oxidative exfoliation, GO displays a new stretching vibration of epoxide at 1257 cm^−1^, the carbonyl peak at 1732 cm^−1^, the broad peak of hydroxyl around 3200 cm^−1^, and the sp^2^ hybridized C=C vibration band at 1626 cm^−1^, definitely confirming the successful synthesis of GO. After the reduction by hydrazine, the epoxide peak at 1257 cm^−1^ was absent and the –OH peak around 3000 cm^−1^ was weakened and much more broad than that of GO, but there still existed a weak peak at 1720 cm^−1^ corresponding to C=O which had an intensity much lower than that of GO. This result indicates that the hydrazine reduction can eliminate most of the oxygenated functionalities such as hydroxyls and epoxides, while leaving a number of carbonyl and carboxyl groups at the edges [27]. The relative intensity ratios of polar groups to C=C vibration (the C=C peak is reasonably used as the reference for the normalization of all peaks because the sp^2^ C=C skeleton is the primary structure for either GO or rGO and also stays relatively more stable during oxidation and reduction compared to those oxidative groups) are shown in Table 3. It is obvious to see the decreased intensity ratios of polar groups to C=C band for rGO compared to GO, indicating the largely reduced oxidation degree after the chemical reduction. Note that high wavenumber shifting of the C=C backbone from 1626 to 1543 cm^−1^ after reduction was due to the transformation from a C-sp^3^ to C-sp^2^ backbone, which was also observed in the literature [28].

The oxygen-containing functional groups of rGO were partially eliminated and nitrogen species were generated simultaneously during the hydrazine reduction process. A new band emerged at 1656 cm^−1^ in the FTIR spectrum of rGO in Figure 3 corresponding to C=N, indicating that covalent bond-forming reactions occurred, giving rise to hydrazone (C=N–NH_2_) [29]. Additionally, the appearance of a new peak at 1193 cm^−1^, which was the characteristic stretching vibration of the C–N bond from aromatic amines, indicates the formation of hydrazide (–CONHNH_2_) obtained from the reaction between hydrazine and carboxyl. The weakened and broadened –OH peak also contributed the amidation process to partially modify the surface functionality from carboxyl (–COOH) to hydrazide (–CONHNH_2_). To confirm this result and to exclude possible adsorption of DMF, GO was treated in the same condition for its reduction but without hydrazine. The FTIR spectra of GO and “treated” GO showed the same ratio for the peak and we could not observe the emergence of peaks associated to nitrogen species. This experiment proves that the DMF was not adsorbed into the graphene. 

**Figure 4:**
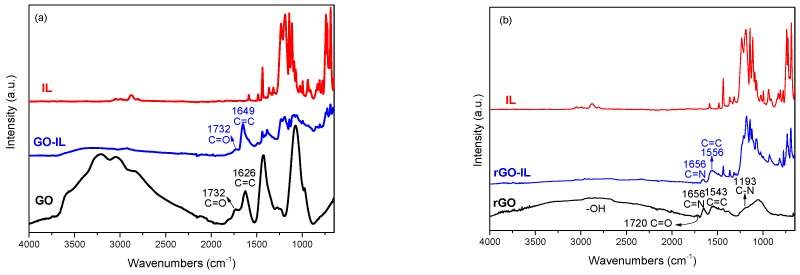
ATR FTIR spectra of GO, IL, GO-IL (**a**) and rGO, IL, rGO-IL (**b**).

The modification of the surface of GO and rGO with ILs can be further confirmed by FTIR Spectra in Figure 4. For both GO-IL and rGO-IL, the characteristic peaks at 1587, 1485, 1437, 1144, 810, 741, 725, and 688 cm^−1^ corresponding to IL alone were found, indicating that ILs were functionalized on the surfaces of GO or rGO. Moreover, the C=C backbone vibration showed an obvious blue-shifting from 1626 to 1649 cm^−1^ for GO-IL in Figure 4a and from 1543 to 1556 cm^−1^ for rGO-IL in Figure 4b, respectively, further corroborating the successful modification of GO or rGO by ILs due to the specific interactions between ILs and GO or rGO.

**Figure 5:**
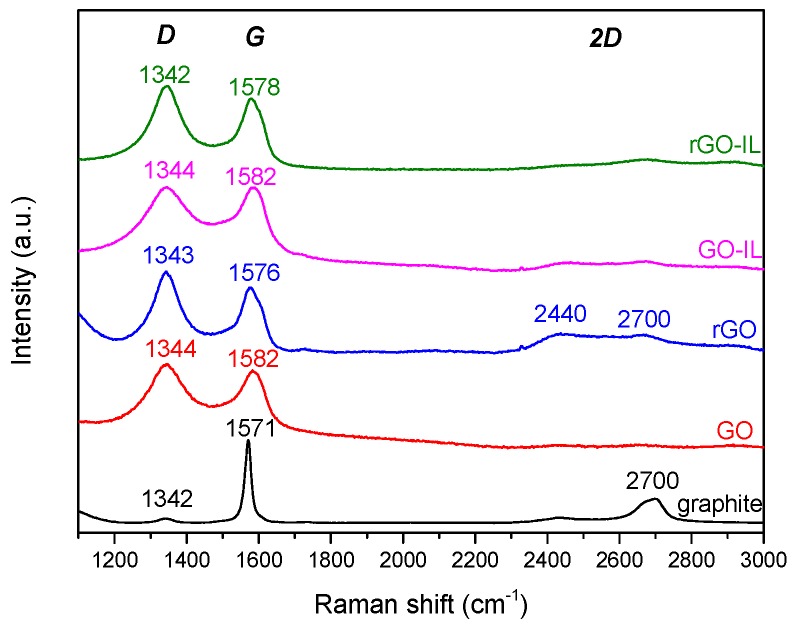
Raman spectra of graphite, GO, rGO, GO-IL, and rGO-IL.

Figure 5 shows the Raman spectra of graphite, GO, rGO, GO-IL, and rGO-IL. The D band at ~1340 cm^−1^ and G band at ~1580 cm^−1^ characteristic of graphene were evidenced. Generally, the D band corresponds to the first-order zone boundary phonons only observed in defected graphene, while the G band is associated with the in-plane optical vibration [30]. The intensity ratio (*I*_D_/*I*_G_) of the D band and G band is usually used to qualitatively characterize the defect density, namely, the lower value of *I*_D_/*I*_G_, the higher perfection of the sp^2^-hybridized structure. A strong G band at 1571 cm^−1^ and a weak D band at 1342 cm^−1^ can be found in the Raman spectrum of graphite with a very low *I*_D_/*I*_G_ value of 0.069, indicating the primary sp^2^-hybridized carbon atoms in graphite without a significant number of defects (summarized in Table 4).

After oxidation, a very obvious D band appeared at 1344 cm^−1^ for GO, whereas the G band of GO broadened and shifted to a higher wavenumber position at 1582 cm^−1^. The increase of the D band demonstrated the introduction of defect-like domains in graphene structures due to the oxidative groups and, thus, the corresponding *I*_D_/*I*_G_ ratio dramatically increased to 1.056. Further increased defect density with the *I*_D_/*I*_G_ ratio of 1.110 was observed after reduction by hydrazine in the Raman spectrum of rGO. Moreover, the G band slightly moved to a lower wavenumber position, which means the recovery of the sp^2^ domain of graphite to a certain extent by eliminating oxidative groups. The increase in the defect density of rGO was ascribed to the fewer in-plane sp^2^ domains upon the incorporation of nitrogen atoms on the edge of rGO after reduction by hydrazine [31]. The Raman spectra of GO-IL and rGO-IL show very similar D and G bands and I_D_/I_G_ ratios very close to those of GO and rGO, respectively, indicating that no further defect structure incorporation in graphene sheets was highlighted after the modification of IL on the surface of GO and rGO.

**Figure 6:**
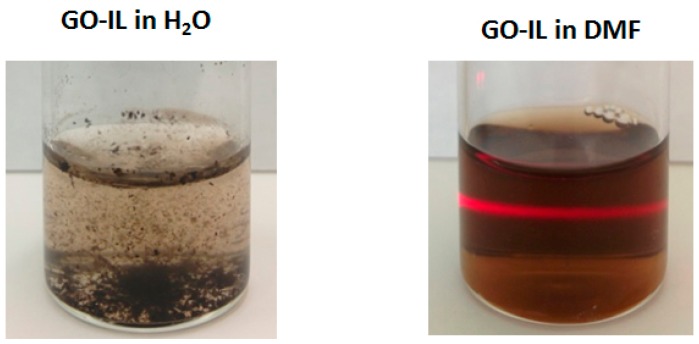
Dispersibility of GO-IL in water and DMF (*c* = 0.5 g/L at room temperature (RT)). Note that GO-IL cannot disperse in water even after 8 h of ultrasonication treatment.

**Figure 7:**
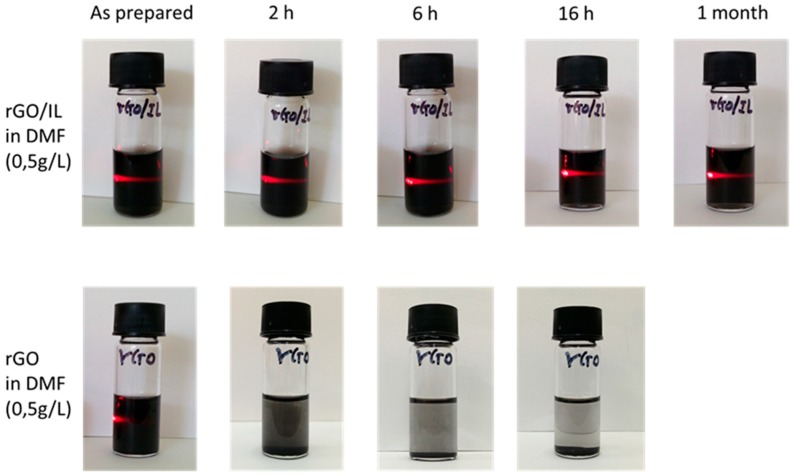
Dispersibility of GO in water and DMF (0.5 g/L at RT).

**Figure 8:**
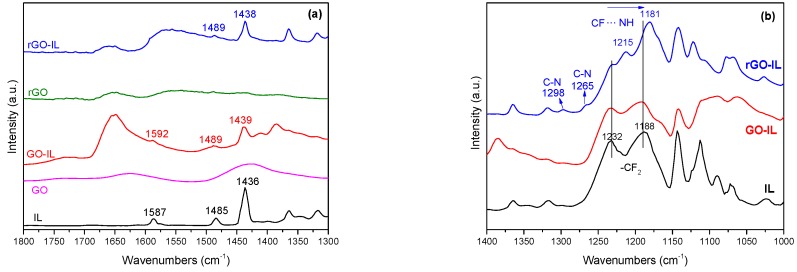
ATR FTIR spectra of IL, GO-IL, and rGO-IL in the range of wavenumbers from 1300 to 1800 cm^−1^ (**a**) and from 1400 to 1000 cm^−1^ (**b**).

**Figure 9:**
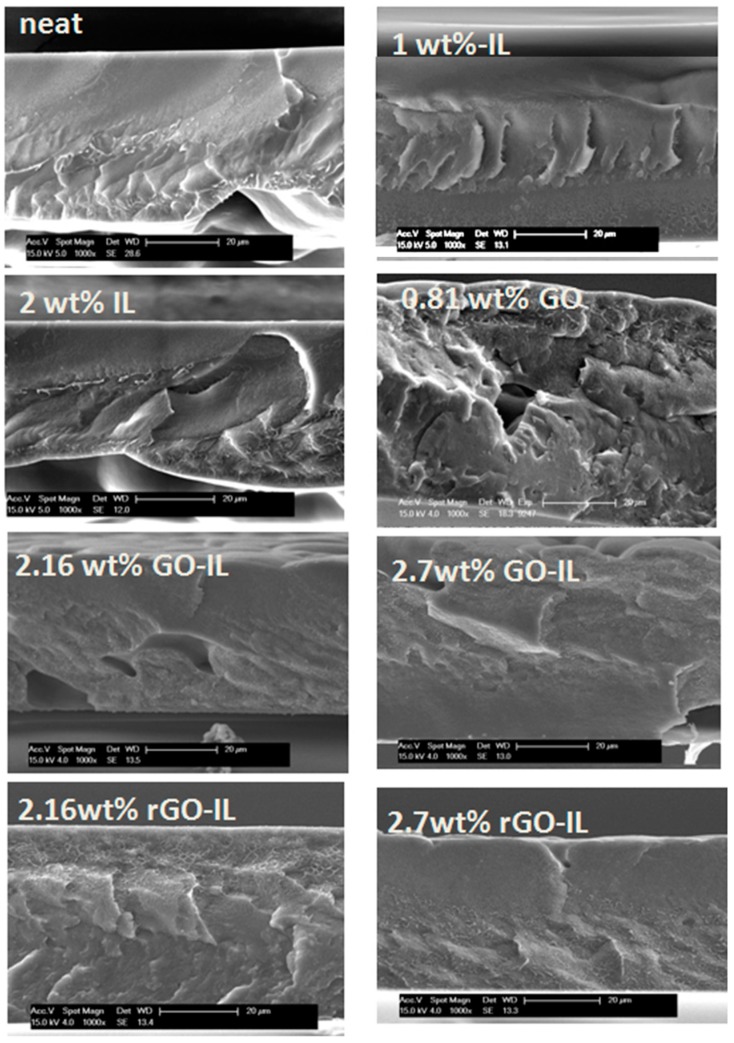
SEM images of fractured cross-sections of composite films containing IL, GO, GO-IL, and rGO-IL.

In addition, another important profile of graphite in the Raman spectrum is the presence of a 2D band at 2700 cm^−1^, which is used to evaluate the structural parameters of the c-axis orientation, since this band is very sensitive to the number of layers (<5 monolayers) and stacking order of the graphite along the c-axis [32]. In Figure 3, Figure 4, Figure 5, Figure 6, Figure 7, Figure 8 and Figure 9, a visible single peak at 2700 cm^−1^ can be found for pristine graphite, indicative of stacking graphitic multilayers. However, after the chemical oxidation process, GO shows no peaks in the 2D band at all because of the predominant structural changes in the graphite lattice due to the formation of different types of oxygenated functional groups intercalated among the graphitic layers at the basal plane and also at the edges, leading to the breaking of the stacking order [33,34]. When GO was reduced by hydrazine, rGO redisplayed a very broad 2D band with a new generated lower-wavenumber peak at 2440 cm^−1^ instead of 2700 cm^−1^ one. This reappearance of a 2D band was due to the recovery of ordered graphitic stacking after eliminating the oxidative groups. Although the reaggregation of graphene sheets occurred for rGO, the number of layers was still lower than five, and the lower-wavenumber shifting indicates that the number of graphitic layers for rGO was lower than that of graphite, which has been accepted in the literature [35]. However, it is interesting to see the dramatic suppression of a 2D band for rGO-IL. This behavior could suggest the successful modification of rGO by IL due to the breaking of the ordered multilayer structure along the c-axis, resulting in random dispersion of graphene sheets thanks to the specific interactions between IL and graphene.

The modification of GO by IL-C8F13 can be easily confirmed by the dispersion of GO-IL in different solvents (see photos in Figure 6). First, the solubility of IL in water and DMF was experimentally compared. Due to the hydrophobic nature of IL containing a fluorinated chain, IL was found to be not soluble in water but in DMF. Second, note that a mixed solvent water/DMF (2/1, v/v) was used to wash out unmodified free IL during the modification because it was found that IL can be dissolved in this mixed solvent but not for GO-IL. After modification, GO-IL decants in water due to the change in the surface profile. It is very preferable that GO-IL can still form a good dispersion in DMF at the nanoscale because DMF is also a good solvent for P(VDF-CTFE) at the concentration used in the following preparation of polymer composites via the solution casting method.

It is well-known that the direct reduction of GO will result in inevitable aggregations due to the loss of polar groups on the surface, which is not required to obtain a good dispersion in the polymer matrix within the cosolvent [36,37]. After modification with IL, rGO-IL showed a very stable colloid dispersion in DMF even over a long time (~1 month), while the counterpart rGO was hard to disperse well, even under the same ultrasonic treatment and decanted only in 1~2 h (see pictures in Figure 7). This imperfect dispersion of rGO in DMF due to the absence of IL modification indicates that rGO cannot be used to prepare composites with P(VDF-CTFE) with a good distribution at the nanoscale. Thus, IL plays a key role in the dispersion of modified rGO in a polar solvent. The perfect dispersion in a cosolvent (DMF) for both filler and polymer matrix was very important to the preparation of a conductive rGO fillers-incorporated polymer matrix P(VDF-CTFE) with a good dispersion. Therefore, only P(VDF-CTFE) composites containing GO, GO-IL, and rGO-IL as fillers were prepared but not for those containing rGO due to the bad dispersion of rGO in cosolvent DMF.

#### 3.1.2. New Interaction Model: Hydrogen Bond C–F∙∙∙H–N between rGO and IL

As we mentioned in the FTIR spectroscopy above, the blue shifting of C=C vibration after IL modification confirms the interaction between IL and graphene which is the driving force to modify the surface of GO or rGO by IL. First, thanks to three phenyls in the cation structure of IL, the π–π or cation–π interaction between IL and GO or rGO is one of the interacting forces [13]. This π–π or cation–π interaction can be reflected by the blue shifting of breathing vibration of benzene rings of IL at 1436, 1485, and 1587 cm^−1^ as shown in Figure 8a to 1439, 1489, and 1592 cm^−1^ for GO-IL, and to 1438 and 1489 cm^−1^ for rGO-IL, respectively. Second, the ionic interaction also contributes to the bonding between GO and IL. It is well-known that an alkaline solution adjusted by NaOH can capture protons of COOH groups on the edge of GO, generating negatively charged carboxylate COO–. Through the following cation-exchange between Na^+^ and cation of IL, IL is thus bonded on the surface of GO via ionic interactions [38]. The ionic interactions between carboxylate and IL can be verified by the relative intensity attenuation of the broad –OH band in COOH groups around 3000 cm^−1^ in Figure 4a after the IL modification onto the surface of GO.

**Scheme 2:**
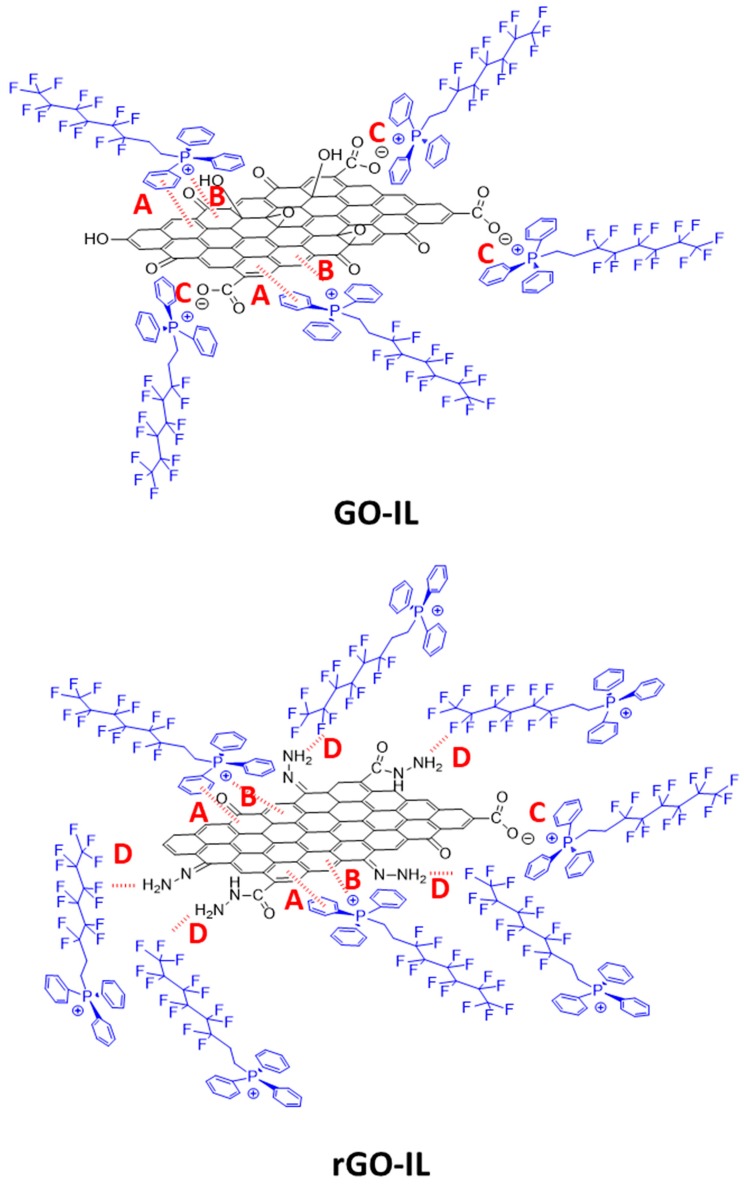
Difference in the interaction model between GO-IL and rGO-IL with ILs: (**A**) π–π interaction; (**B**) cation–π interaction; (**C**) ionic interaction; (**D**) hydrogen bonding interaction.

However, once the chemical reduction of GO by hydrazine occurs, the interaction model between rGO and IL largely deviates from that between GO and IL because of the partially remaining oxygen functional groups and nitrogen doping of the rGO surface after the oxygen removal process by hydrazine. On the one hand, as shown in Figure 4b, the very broad –OH band in COOH groups in the range of wavenumbers from 3300 to 3500 cm^−1^ in rGO became hardly observable in rGO-IL, indicating that there are some ILs interacting with residual COO– groups at the edge of rGO via ionic interaction model. On the other hand, as we mentioned above, the surface of rGO became nitrogen doped: hydrazone and hydrazide were produced during the treatment of hydrazine reduction; these two nitrogen-containing species can also be obtained during the preparation of rGO-IL. The newly generated hydrazone and hydrazide were expected to be responsible for the C=N vibration at 1656 cm^−1^ (in Figure 4b) and C–N bonds at 1265 or 1298 cm^−1^ observed in FTIR spectra of rGO-IL in Figure 8b. Furthermore, we carried out a parallel experiment to prove that IL-C8F13 was chemically stable under the reduction conditions by hydrazine (95 °C for 3 h in DMF with the same concentration), which was confirmed by the identical NMR spectra before and after hydrazine treatment (see the ^1^H, ^13^C, ^19^F, and ^31^P NMR spectra in Figure S2). Therefore, the newly formed NH-containing groups after reduction was considered to be involved in the newly generated H-bond interaction with the C–F bond of fluorinated IL, and this type of N–H∙∙∙F–C H-bonding interaction was confirmed by Chaudhari et al. [39]. Moreover, two strong vibration bands at 1232 and 1188 cm^−1^ assigned to the asymmetric and symmetric stretch of –CF_2_, respectively, [40] of IL alone show no change in GO-IL but a very obvious red-shifting to 1215 and 1181 cm^−1^, respectively, for rGO-IL (see Figure 8b), confirming the H-bonding interaction of N–H…F–C experimentally. Consequently, the increased ratio of this new H-bonding model was accompanied with the decrease in the ionic interaction in the preparation of rGO-IL in which the –COOH groups were partially reduced and graphene surfaces became nitrogen doped. It was the newly generated H-bonding interaction between rGO and IL combined with the original ionic interaction that contributed to the reliable and stable modification of IL on the surface of rGO, which cannot be removed by washing many times. The different interaction models of GO-IL and rGO-IL are presented in Scheme 2. In addition, the difference in the interaction models between GO-IL and rGO-IL may explain the phenomenon that the corresponding IL absorption peaks of rGO-IL were more obvious than those of GO-IL from the FTIR results in Figure 4. The reduction treatment by hydrazine converted the carbonyl and carboxyl groups into hydrazone and hydrazide, respectively, which increased the interacting sites on the surface of rGO with IL, while for GO, only carboxyl groups on the edge of GO could supply interacting sites with IL.

### 3.2. Characterizations of the Composites

#### 3.2.1. Dispersion Morphology

**Figure 10:**
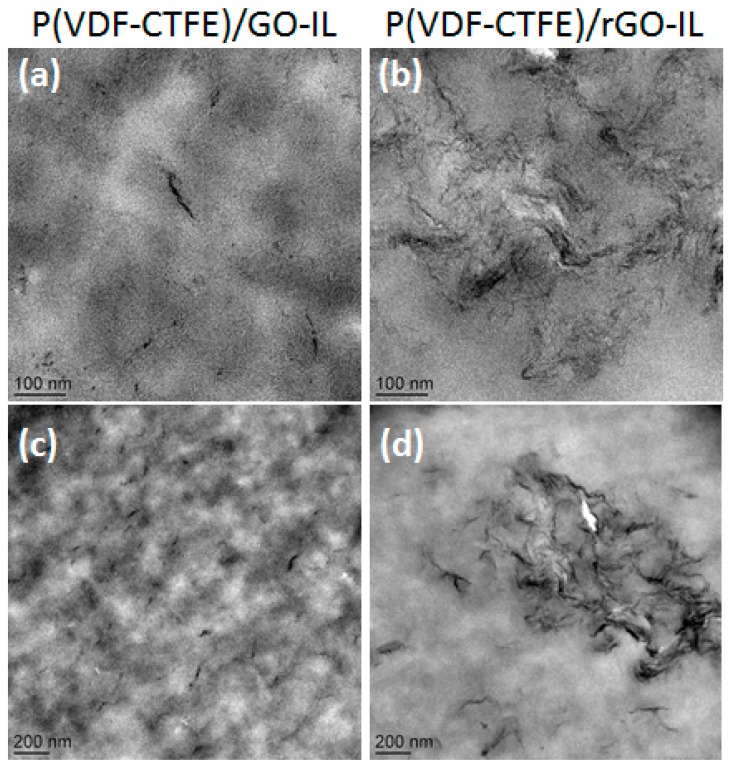
TEM images of poly(vinylidene fluoride-co-chlorotrifluoroethylene) (P(VDF-CTFE))/GO-IL (**a**,**c**) and P(VDF-CTFE)/rGO-IL (**b**,**d**) with 2.70 wt% of fillers.

The fractured cross-section morphologies of composite materials containing IL, GO, GO-IL, and rGO-IL by SEM are shown in Figure 9. The tortuous morphology of each sample reveals a plastic deformation witnessing of the excellent adhesions of fillers with polymer matrix. Any fillers aggregates cannot be detected, highlighting a rather homogeneous dispersion state. However, we cannot see graphene sheets within polymer matrix by fractured cross-section images of SEM. To further evaluate the dispersion and the distribution state of GO-IL and rGO-IL within polymer matrix, a TEM study was employed. In Figure 10a,c, it is apparent that the GO-IL sheets were individually and randomly well dispersed in the P(VDF-CTFE) matrix, whereas some aggregates of rGO-IL were observed within the polymer matrix from the TEM images shown in Figure 10b,d for composites with rGO-IL.

The formation of the enriched rGO-IL zones within polymer composites may be due to the reduction of oxidative groups from GO-IL, which, to some extent, induces more restacking of graphene layers among which there are almost no oxidative groups. Moreover, it could be ascribed to the change in the interaction model between graphene and IL during the reduction process. As mentioned before, a new H-bonding interaction model of rGO-IL through N–H…F–C association is different from that of GO-IL. In this way, when rGO-IL fillers are incorporated in the polymer matrix, for a given rGO-IL sheet, the IL bonded on the surface of one rGO sheet can also be involved in other H-bonding interactions with neighboring graphene sheets, resulting in a morphology with enriched rGO-IL clusters within the polymer matrix shown in the TEM images.

#### 3.2.2. Crystalline Phase: Total γ-Phase Transformation

**Figure 11:**
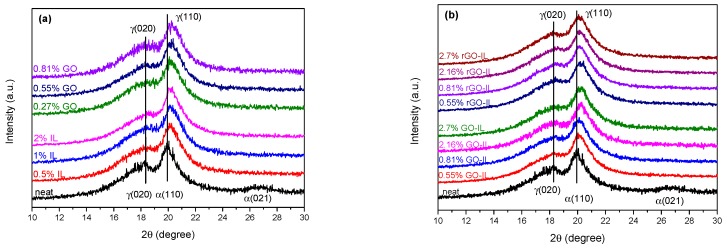
XRD patterns of neat P(VDF-CTFE) and composites with IL and GO (**a**) and GO-IL and rGO-IL (**b**).

**Figure 12:**
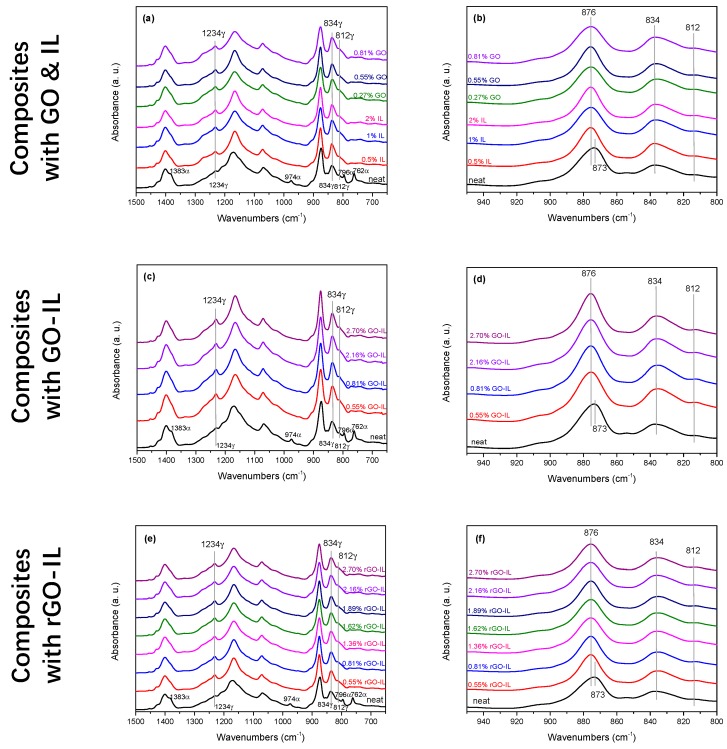
ATR FTIR spectra of neat P(VDF-CTFE) and composites with IL, GO (**a**,**b**); GO-IL (**c**,**d**); and rGO-IL (**e**,**f**).

It is well known that polar crystalline phases (β- or γ-phase) are required for electroactive properties and more specifically piezoelectric and ferroelectric properties [9,41]. The intermediate polar γ-phase pays attention to ferroelectric properties, as described by Barrau el al [42]. Moreover, a higher breakdown strength and higher discharge energy density are observed for the γ-phase compared to the β-phase [43]. The XRD pattern of neat P(VDF-CTFE) film from DMF in Figure 11 shows two characteristic diffraction peaks at 2θ = 19.9° and 26.6° assigned to (110) and (021) reflections of α-phase crystal, respectively, and the third peak at 2θ =18.3° corresponds to (020) plane of the γ-phase. Moreover, the corresponding FTIR spectrum in ATR mode of neat P(VDF-CTFE) (Figure 12) displayed the characteristic absorption peaks of α-phase at 762, 796, 974, and 1383 cm^−1^, and also the characteristic γ-phase peaks at 812, 834, and 1234 cm^−1^. The XRD results combined with the FTIR results definitely corroborate the presence of α- and γ-crystal mixture in neat copolymer. The crystalline pattern of P(VDF-CTFE) from DMF differs from the one obtained from methyl ethyl ketone (MEK), in which the neat copolymer presented only α-phase because of the relatively lower polarity of MEK. However, with adding all fillers, i.e., IL-C8F13, GO, GO-IL, and rGO-IL in the polymer matrix, the non-polar α-phase was completely transformed into a polar γ-phase regardless of the type and quantity of fillers incorporated in the polymer matrix. This entire transformation behavior from mixed α- and γ-phase into a pure γ-phase was confirmed by the disappearance of diffraction peaks of the α-phase and the observation of another new γ-phase peak at 2θ = 20.1° ascribed to the γ(110) crystal plane in XRD patterns (shown in Figure 11), and also by the vanishing of the α-phase characteristic peaks at 762, 796, 974, and 1383 cm^−1^ but the emergence of typical bands of the γ-phase at 812, 834, and 1234 cm^−1^ in FTIR spectra (shown in Figure 12a,c,e). From XRD and FTIR results, it is easily found that both GO and IL can induce total crystalline-phase transformation from α-phase to γ-one due to the 2D template effect of GO and dipolar interaction of IL with polymer chains, respectively, on the chain configuration of P(VDF-CTFE) to stabilize more TTT sequence [12,13,44,45]. Consequently, combining the crystal transformation effect induced from both graphene and IL, the fillers of GO-IL and rGO-IL can also be induced thorough transformation of α-phase to γ-phase.

Moreover, in the zoomed FTIR spectra of all composites in the range of 800 to 900 cm^−1^ in Figure 12b,d,f, the peak at 873 cm^−1^ corresponding to amorphous –CF_2_–CH_2_– bending vibration, which is insensitive to crystalline phase transformation in P(VDF-CTFE) [46], shows a blue shift to 876 cm^−1^, indicating the interactions of the polymer matrix with the fillers including IL, GO, GO-IL, and rGO-IL. The interaction of IL with copolymer was ascribed to dipolar interactions of ions of ILs and C–F dipole from a fluorinated short chain of cations with dipoles of C–F or C–Cl in the P(VDF-CTFE) copolymer. Note that the peak shifts of two vibration bands at 1232 and 1188 cm^−1^ assigned to the –CF_2_ asymmetric and symmetric stretch in IL cations (if C–F dipoles in fluorinated short chains of cations in IL interact with polymer chains) cannot be seen in composites containing IL, GO-IL, or rGO-IL because of the superimposition of peaks in this zone for a fluorinated copolymer matrix. The interactions of GO with polymer can be explained by the presence of oxygenated functional groups on the surface of GO with polymer matrix. Therefore, the dipolar interactions from both IL and GO or rGO with a polymer matrix induce a stronger interfacial interaction between the GO-IL or rGO-IL and the P(VDF-CTFE), subsequently stabilizing the dispersion of GO-IL or rGO-IL in a polymer matrix.

#### 3.2.3. Crystallization Behavior: Heterogeneous Nucleation Effect

It is well known that the fillers can significantly influence the crystallization behavior of a semi-crystalline polymer matrix. The values of *T*_c_ (note that the values of T_c_ from the first cooling run are the same as those from the second cooling run), *T*_m_ and melting enthalpy (Δ*H*) (the first and second heating run) of neat P(VDF-CTFE) and composites containing either IL-C_8_F_13_ alone or modified graphene from DSC thermograms (see Figure S3 in the Supplementary Materials) are summarized in Table S1 of the Supplementary Materials. Note that the values of *T*_m_ and Δ*H* from the first heating run vary from those of the second heating run. It could result that the first heating run reflects the properties of as-prepared films, namely, the crystallization process from solution during the film formation process. The results from the second heating run indeed illustrate the crystallization process from melting without the heat history of samples.

By increasing the amount of IL-C8F13 from 0.5 to 2 wt% within P(VDF-CTFE) matrix, *T*_c_ displays no obvious change, while *T*_m_ from the second heating run and melting enthalpy in both first and second heating run show an increasing effect compared to the matrix. However, an opposite depression behavior (deceased *T*_c_, *T*_m_, and melting enthalpy) has been demonstrated for the counterpart IL-C18 in which a C18 alkyl substituent as well as three phenyls are on the cation [47]. This difference can be ascribed to the functionality of the cation structure: a fluorinated C8F13 substituted chain (IL-C8F13) versus an alkyl C18 (IL-C18) chain. A fluorinated short chain with regularly repeated C–F dipoles can be seen as a short-range order structure, and it can facilitate the orientation of polymer chains, inducing a more perfect arrangement of polymer chains in crystallites with elevated *T*_m_ through dipole–dipole interaction with P(VDF-CTFE). This interaction should be overcome during the melting process, thus increased values of melting enthalpy were observed. Differing from IL-C8F13, IL-C18 possesses a long alkyl chain and because of the absence of repeat C–F units, it cannot supply the regular range order structure prompting the orientation of polymer chains to form crystals, thus, IL-C18 has been seen as an obstacle in the crystallization of P(VDF-CTFE), leading to the depression of crystallization behavior.

For GO-incorporated composites, the values of *T*_c_ also show no clear change, same as IL-C8F13, suggesting no heterogeneous nucleation effect which is distinguished to the extensively demonstrated 2D template heterogeneous nucleation effect of graphene on many semi-crystalline polymers [48]. This behavior can be rationally explained by the relatively weak oxidative groups on the surface of GO in our system which cannot provide enough interaction to absorb polymer chains on the 2D template structure and, thus, cannot become a precursor to induce crystal growth. Besides the unchanged *T*_c_, the values of *T*_m_ of GO/P(VDF-CTFE) from either first or second heating run are almost similar to the neat one, indicating no promotional effect on the perfect arrangement of polymer chains in crystals due to the random existence of oxidative groups on the surface unlike IL-C8F13 with a so-called short-range order structure. Although the relatively weak interaction and random arrangement of oxidative groups of GO result in no significant effect on *T*_c_ and *T*_m_, the interaction between oxidative groups of GO and dipoles of P(VDF-CTFE) also contributes to the energy requirement needed to disrupt the crystallites with increased values of melting enthalpy compared with a neat one.

By combining both GO and IL or rGO and IL, it is remarkable to see the significant enhancement of T_c_, indicating the strong nucleation effect for P(VDF-CTFE) crystallization in the presence of GO-IL and rGO-IL. Ionic liquid-modified graphene is, therefore, acting as nucleating agent to cause easier nucleation and crystal growth by combining the 2D template effect of graphene for absorption of polymer chains and fluorinated short chain of IL-C8F13 with short-range order structure and relatively strong dipole–dipole interaction. This synergistic effect also gave rise to elevated *T*_m_ values from the second heating run for GO-IL/P(VDF-CTFE) and rGO/P(VDF-CTFE) composites compared to the neat one due to the heterogeneously nucleated and more perfect crystals. However, note that the values of *T*_m_ of composites containing IL, GO-IL, and rGO-IL from the first heating run showed comparable values with the neat P(VDF-CTFE). This behavior may be ascribed to the difference in the crystallization process, i.e., IL, GO-IL, and rGO-IL fillers do not affect the perfection of crystalline domain or very weakly only through evaporating solvent to obtain films. Concerning the values of melting enthalpy, GO-IL/P(VDF-CTFE) also showed a greater energy requirement than a neat one in the full melting process to destroy the interactions, including (1) IL with polymer and (2) oxidative groups with polymer. However, due to the reduction of partial oxidative groups for rGO-IL, the contribution from the interaction of oxidative groups with polymer was reduced, leading to a slight decrease in the melting enthalpy for composites of rGO-IL/P(VDF-CTFE).

#### 3.2.4. Relaxation Behavior

**Figure 13:**
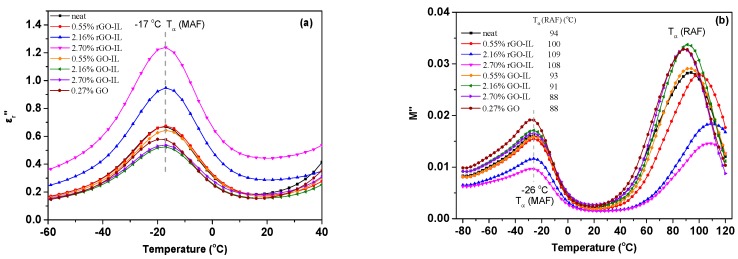
Imaginary part of relative permittivity (ε_r_”) (**a**) and dielectric modulus (M”) (**b**) as a function of the temperature of neat P(VDF-CTFE) and composites containing GO, GO-IL, and rGO-IL (*f* = 1 kHz, the first heating run). Note that the peak position and intensity from the first heating run is the same as the one from the second heating run).

Dielectric spectroscopy and dynamic mechanical analysis were carried out to study relaxations of the fluorinated matrix and to evaluate the impact of the different fillers to the amorphous phase. The different effects of GO-IL and rGO-IL fillers on the polymer chain segmental relaxation can be reflected by dielectric analysis in Figure 13. For all composites containing GO, GO-IL, and rGO-IL, the values of *T*_α_ at −17 °C corresponding to the relaxation in the mobile amorphous fraction (MAF) were the same as the neat P(VDF-CTFE) determined from the imaginary part of relative permittivity (ε_r_”) as a function of temperature at 1 kHz in Figure 13a, indicating that all fillers including GO, GO-IL, and rGO-IL had no influence on the free volume of P(VDF-CTFE) in the MAF region. Moreover, the consistent values of *T*_α_ (MAF) for all composites compared to the P(VDF-CTFE) matrix were also observed in the plot of imaginary part of dielectric modulus (M”) as a function of temperature in Figure 13b in which the relaxation occurred at −26 °C. In addition, the DMA results shown in the Supplementary Materials also demonstrated this behavior. The same values of *T*_α_ at −36 °C are shown for both the neat matrix and all composites containing IL (Supplementary Materials Figure S4a), GO (Figure S4b), GO-IL (Figure S4c), and rGO-IL (Figure S4d). This behavior of unchanged *T*_α_ for all composites compared to the neat P(VDF-CTFE) matrix indicates that the fillers have no plasticization effect on the P(VDF-CTFE) matrix.

Nevertheless, the second *T*_α_ peak localized at higher temperature corresponding to defect movements in the rigid amorphous region (RAF), regarded as the amorphous/crystalline interface with relatively immobile polymer chains confined among crystallites [49], showed shifting for all composites. The shift of relaxation peak in the RAF region for all the composites can be explained by the nucleation effect of GO-IL or rGO-IL which induced the crystal growth, and, thus, influenced the chain segmental relaxation in this area. The relaxation of neat P(VDF-CTFE) in the RAF region occurs at 94 °C, while for composites containing GO and GO-IL, the values move to lower temperature. This lower T shift can be ascribed to the increased free volume in the RAF region. The interaction between amorphous polymer chains in the RAF region and either oxidative groups or IL on the surface of GO improves the dispersion of fillers in the matrix and also weakens the inter-chain affinity of P(VDF-CTFE), which facilitates the chain segmental movement with a lower T shift of relaxation. However, it is interesting to find an opposite shift trend (higher T shift) for all composites containing rGO-IL. The reduced free volume of polymer chain in the RAF region induced by rGO-IL accompanied with higher T shift of relaxation could be due to the different dispersion morphologies induced by GO-IL and rGO-IL within the polymer matrix. As shown in Figure 10, the composites with rGO-IL exhibited a morphology with rGO-IL-enriched clusters within the polymer matrix which, to some extent, constrained the polymer chains of P(VDF-CTFE) copolymer, leading to an increased density in the amorphous zone in the RAF. Thus, the chain segmental motion was hindered in the RAF, causing a difficulty in the relaxation process [45].

#### 3.2.5. Electrical Conductivity of P(VDF-CTFE)/GO Composites

**Figure 14:**
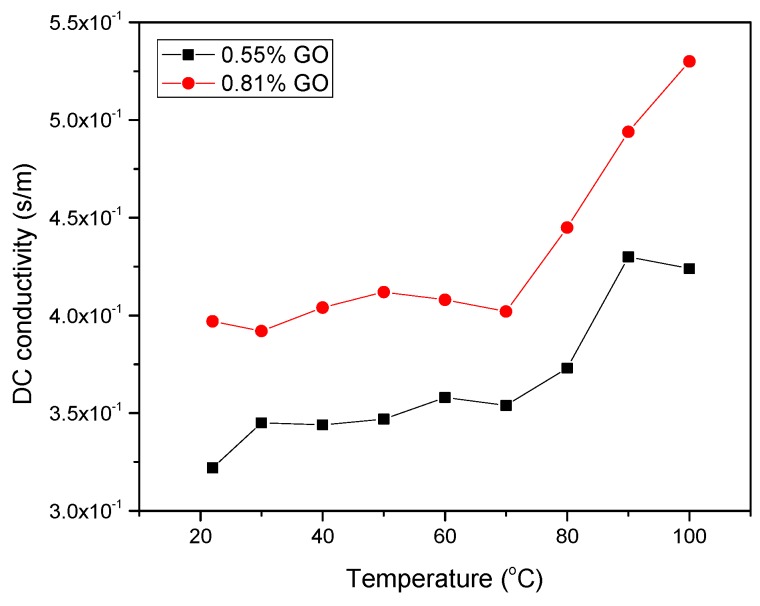
Direct current (DC) conductivity as a function of temperature of P(VDF-CTFE)/GO.

Figure 14 shows the DC conductivity of P(VDF-CTFE)/GO as a function of temperature. It is obvious to see the conductive nature of composites containing GO alone from 0.27 wt% (the composite with 0.27 wt% GO act as a dielectric material, data not shown). The values of DC conductivity at RT with 0.55 and 0.81 wt% of GO are 3.22 × 10^−1^ and 3.97 × 10^−1^ S/m, respectively. The DC conductivity of P(VDF-CTFE)/GO shows temperature stability in the range from RT to 70 °C, while starting from 70 °C, the DC conductivity presents a relatively sharp increase. This behavior is potentially related to the *T*_α_ (RAF) relaxation at around 70~80 °C for neat P(VDF-CTFE).

#### 3.2.6. Dielectric Properties of P(VDF-CTFE)/IL-Modified Graphene Composites

**Figure 15:**
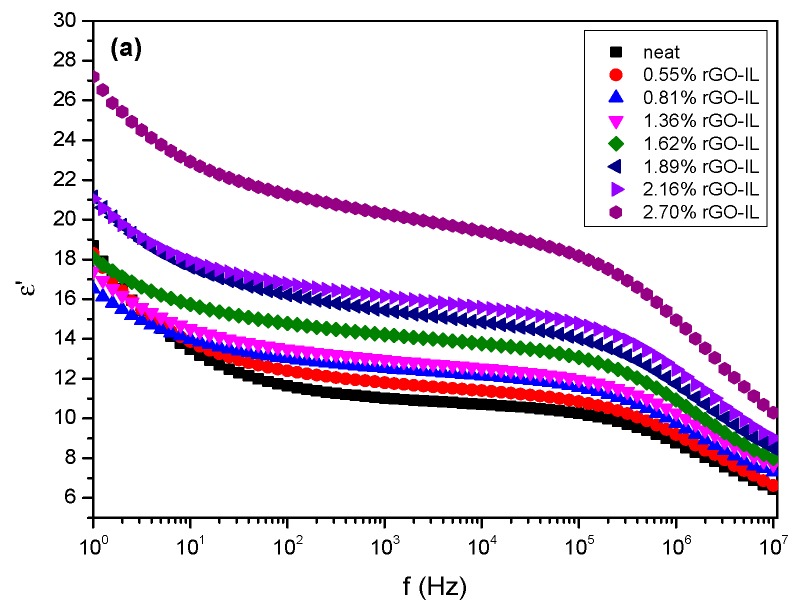

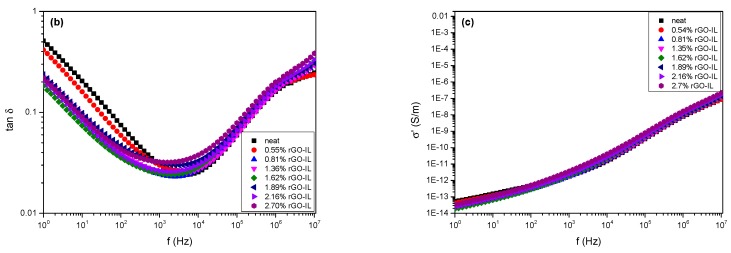
Relative dielectric permittivity (ε_r_’) (**a**), loss tangent (tan δ) (**b**), and AC conductivity (σ’) (**c**) of P(VDF-CTFE)/rGO-IL composites as a function of frequency at RT with different compositions.

With the attachment of ILs onto the surface of graphene (rGO or GO), the composites act as dielectrics. The curves of dielectric permittivity (ε’), loss tangent (tan δ), and AC conductivity (σ’) as a function of frequency at RT for P(VDF-CTFE)/rGO-IL composites with different compositions are plotted in Figure 15. It is obvious to see the gradually increasing behavior of permittivity with increasing rGO-IL loading content, while the dielectric losses have almost no change in the whole frequency range, whose values at 1 kHz are listed in Table 5. For example, the permittivity at 1 kHz of composite with 2.70% of rGO-IL increased up to 20.3 compared with that of 11.0 for the neat one, while tan δ was 0.032 which was almost the same as the value of the neat one (tan δ = 0.030). The increase in dielectric constant was attributed to the good dispersion of rGO with the help of IL and the subsequent formation of so-called “microcapacitors” in the P(VDF-CTFE) matrix, which has been widely reported in the literature [7,50,51,52,53].

Moreover, the formation of clusters, i.e., the zones rich in rGO-IL shown in Figure 10b,d, which exhibited a “network-like” morphology within the polymer matrix may be the second driving force to induce a regular increase of permittivity for P(VDF-CTFE)/rGO-IL. As a comparison, the permittivity of composites containing GO-IL displayed an irregular evolution with the increase of the contents of fillers (see Figure S5), and the permittivity of composites with GO-IL was similar to the neat matrix. The phenomenon could be due to the random dispersion morphology within polymer matrix with GO-IL, where GO-IL fillers are separated from each other in the polymer matrix and the electrical properties of the composites are dominated by the matrix. Thus, the difference in the permittivity evolution behavior using rGO-IL and GO-IL demonstrates the different effect of dispersion morphology of IL-modified graphene used as fillers on the enhancement of permittivity of composites.

As mentioned above, the composites with GO displayed conductive characteristics. However, when ILs are anchored onto the surface of graphene through ionic and H-bonding interactions, the composites with rGO-IL and GO-IL showed dielectric behaviors. In this way, ILs not only play a role as interfacial agents to facilitate the dispersion of graphene in polymer matrix, but could also act as isolating shells to prevent the direct contact of the conductive graphene sheets, resulting in quite low dielectric losses for the composites with graphene compared to a neat matrix. Although the P(VDF-CTFE)/IL-modified graphene composites displayed a relatively high permittivity and a low dielectric loss compared with the neat polymer matrix, they can easily breakdown under a high electrical field (lower than 100 V/µm), which is detrimental to the application of this composite material.

For this type of conductive filler-based composites, accompanied with the increase in the dielectric constant, the breakdown strength simultaneously decreased. When the filler content approached the percolation threshold, the dielectric loss was normally high and the breakdown strength was also reduced. It is reported that P(VDF-TrFE-CTFE)/carbon black nanocomposites via a simple solution exhibit excellent mechanical properties and dielectric properties with a dielectric permittivity of 140 and a low dielectric loss of 0.05 at 100 Hz with an *f*_c_ = 4.68 wt% [54]. Nevertheless, the breakdown strength decreased simultaneously due to the enhanced local electric field induced by the thin insulating layer between CB fillers, which is not desirable for practical applications. Although we are facing some issues to utilize well these kinds of composites for applications, it is still promising to bring out the potentials in the high dielectric constant of these conductive filler-based composites but it remains to find an efficient way to reduce the dielectric loss and maintain high dielectric breakdown strength.

Based on the considerations above, it would be interesting to pursue and to complete this work according to various directions. For example, the solution casting strategy to prepare composite films usually produces a porous and defective structure within films due to the high evaporating temperature used for removing solvent DMF, which dramatically decreases the breakdown strength. This issue could be overcome by using melting blending methods such as hot-pressing and melting extrusion.

## 4. Conclusions

In conclusion, a task-specific IL, IL-C8F13 with the cation structure combining three phenyls and a short fluorinated chain, was successfully synthesized and non-covalently modified on the surface of graphene (GO or rGO) due to the specific interactions between IL and GO or rGO. In particular, the H-bonding interaction of N–H∙∙∙C–F of IL with rGO, because of the nitrogen-doped surfaces of rGO by hydrazine reduction, emerged. These IL-modified graphene fillers were then incorporated into the P(VDF-CTFE) matrix to prepare P(VDF-CTFE)/graphene composite materials in which IL played a key role in making compatible graphene fillers and a matrix at the interface among them. The composites showed tortuous morphologies on fractured cross sections thanks to the good dispersion and excellent adhesion of fillers in polymer matrix. The IL-modified graphene fillers worked as heterogeneous nucleating agents for the polymer matrix with increased T_c_ due to the synergistic effects of fluorinated IL and graphene, and they also facilitated the total transformation of γ-phase which is interesting for piezoelectric or ferroelectric properties of fluorinated composites. More interestingly, rGO-IL imposed different effects on crystallization behavior, segmental chain relaxation behavior, and subsequent dielectric properties of composite materials compared to GO-IL because of different interactions of IL with GO or rGO. Finally, it was noticeable that these P(VDF-CTFE)/IL-modified graphene composites exhibited relatively high dielectric permittivity and low dielectric loss, e.g., the relative permittivity at 1 kHz of composite with 2.70% of rGO-IL increased up to 20.3 compared with that of 11.0 for the neat matrix, while tan δ was 0.032 which was almost the same as the value of the neat matrix (tan δ = 0.030). At the same time, the dielectric breakdown strength was dramatically reduced.

## Figures and Tables

**Table 1:** Different compositions of P(VDF-CTFE) composites processed in this work.

Sample	Filler Content (wt%)						
P(VDF-CTFE)/IL	0.5	1	2	–	–	–	–
P(VDF-CTFE)/GO	0.27	0.55	0.81	–	–	–	–
P(VDF-CTFE)/GO-IL	0.27	0.55	0.81	1.08	1.36	2.16	2.70
P(VDF-CTFE)/rGO-IL	0.55	0.81	1.36	1.62	1.89	2.16	2.70

**Table 2:** Summary of parameters from XRD patterns.

Sample	2θ (°)	*d* (Å)	β (°)	*t* (Å)	*n*
graphite	26.5	3.4	0.15	538.3	158.3
GO	9.8	9.0	2.19	36.0	4.0
rGO	24.5	3.6	5.70	14.1	3.9
GO-IL	17.8	5.0	5.63	14.1	2.8

**Table 3:** Intensity ratios of polar groups to C=C band for GO and rGO.

Sample	–OH/C=C	C=O/C=C
GO	3.9	2.2
rGO	1.8	0.2

**Table 4:** Raman shifts of the D band, G band, and 2D band for graphite, GO, rGO, GO-IL, and rGO-IL.

Sample	D Band (cm^−1^)	G Band (cm^−1^)	*I*_D_/*I*_G_	2D Band (cm^−1^)
graphite	1342	1571	0.069	2700
GO	1344	1582	1.056	–
rGO	1343	1576	1.110	2440, 2700
GO-IL	1344	1582	1.003	–
rGO-IL	1342	1578	1.113	–

**Table 5:** Relative dielectric permittivity (ε_r_’) and loss tangent (tan δ) of composites with different rGO-IL loadings at 1 kHz and RT.

wt%	0	0.55%	0.81%	1.36%	1.62%	1.89%	2.16%	2.70%
**ε_r_’**	11.0	11.8	12.5	13.0	14.2	15.4	16.1	20.3
**tan δ**	0.030	0.030	0.024	0.026	0.025	0.032	0.026	0.032

## Supplementary Materials

The following are available online at https://www.mdpi.com/2079-4991/9/8/1181/s1, Figure S1: ^1^H NMR spectra of deuterated acetone (*d*_6_-acetone) solvent (a), P(VDF-CTFE)/GO-IL (0.55 wt%) (b) and P(VDF-CTFE)/GO (0.27 wt%) composites in *d*_6_-acetone. Note that the signal of DMF in *d*_6_-acetone at around 7.96 ppm corresponding to (CH_3_)_2_N-C(O)H was not visible in the spectra of P(VDF-CTFE)/GO-IL and P(VDF-CTFE)/GO composites, suggesting total removing of DMF using the drying conditions: first dried at 60 °C in air oven overnight, and then under vacuum at 80 °C for 48 h. Figure S2: ^1^H, ^13^C, ^19^F, and ^31^P NMR spectra of IL-C8F13 before (upper) and after (lower) hydrazine treatment. Figure S3: DSC thermograms of neat P(VDF-CTFE) and its composites containing GO, GO-IL, and rGO-IL during the first and second heating runs and the first cooling run. Figure S4: Dynamic mechanical loss tangent (tan δ) as a function of temperature for neat P(VDF-CTFE) and composites containing IL (a), GO (b), GO-IL (c), and rGO-IL (d). Figure S5: Relative dielectric permittivity (ε_r_’) (a), loss tangent (tan δ) (b), and AC conductivity (σ’) (c) of P(VDF-CTFE)/GO-IL composites as a function of frequency at RT with different compositions. Table S1: *T*_c_, *T*_m_, and Δ*H* of neat P(VDF-CTFE) and composites from DSC thermograms.
