# Supplementary material for: The Role of Fluorinated IL as an Interfacial Agent in P(VDF-CTFE)/Graphene Composite Films
Source: Nanomaterials (Basel). 2019 Aug 19;9(8):1181. doi: 10.3390/nano9081181 (PMC6724409; doi:10.3390/nano9081181)
Supplement: Supplementary file 1 [file nanomaterials-09-01181-s001.pdf]

Supplementary Materials

The Role of Fluorinated IL as an Interfacial Agent in P(VDF-CTFE)/Graphene Composite Films

Jing Yang, Sébastien Pruvost *, Sébastien Livi and Jannick Duchet-Rumeau *

Laboratory of polymer materials Engineering, UMR CNRS 5223, INSA Lyon, University of Lyon, 69621 Villeurbanne, France

* Correspondence: sebastien.pruvost@insa-lyon.fr (S.P.); jannick.duchet@insa-lyon.fr (J.D.-R.); Tel.:+33-04-72438291 (S.P.); +33-04-72438548 (J.D.-R.)

1. No residual DMF in the resulting composite films

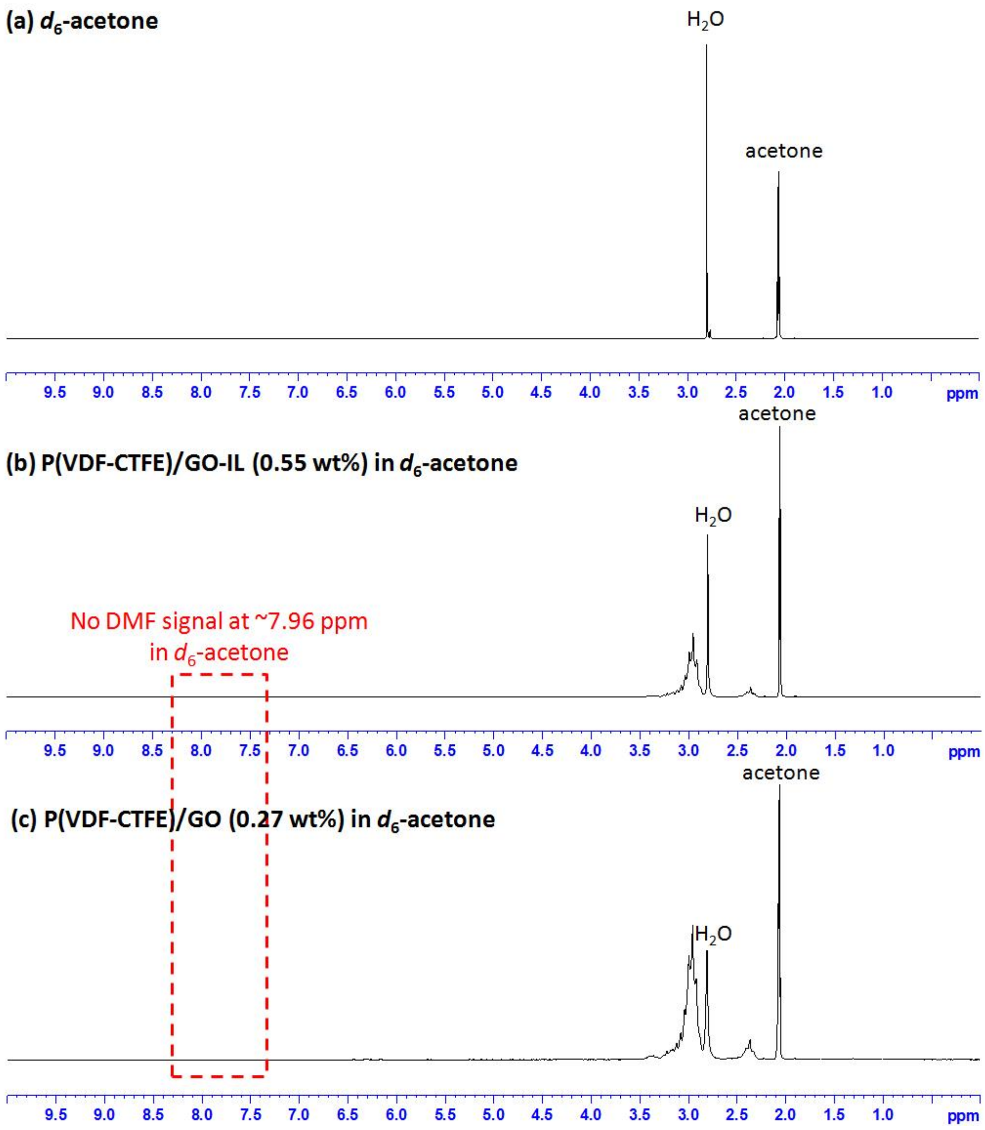

Figure S1. ^{1}H NMR spectra of deuterated acetone (d_6-acetone) solvent (a), P(VDF-CTFE)/GO-IL (0.55 wt%) (b) and P(VDF-CTFE)/GO (0.27 wt%) composites in d_6-acetone. Note that the signal of DMF in d_6-acetone at around 7.96 ppm corresponding to $(CH_3)_2N$-C(O)H (*J. Org. Chem.* **1997**, *62*, 7512-7515) is not visible in the spectra of P(VDF-CTFE)/GO-IL and P(VDF-CTFE)/GO composites, suggesting total removing of DMF by using the drying conditions: first dried at 60 °C in air oven overnight, and then under vacuum at 80 °C for 48 h.

2. Stability of IL-C8F13 during the chemical reduction by hydrazine

The IL-C8F13 is chemically stable under the reduction conditions by hydrazine (95 °C for 3 h in DMF), which can be confirmed by NMR spectra of IL before and after hydrazine treatment.

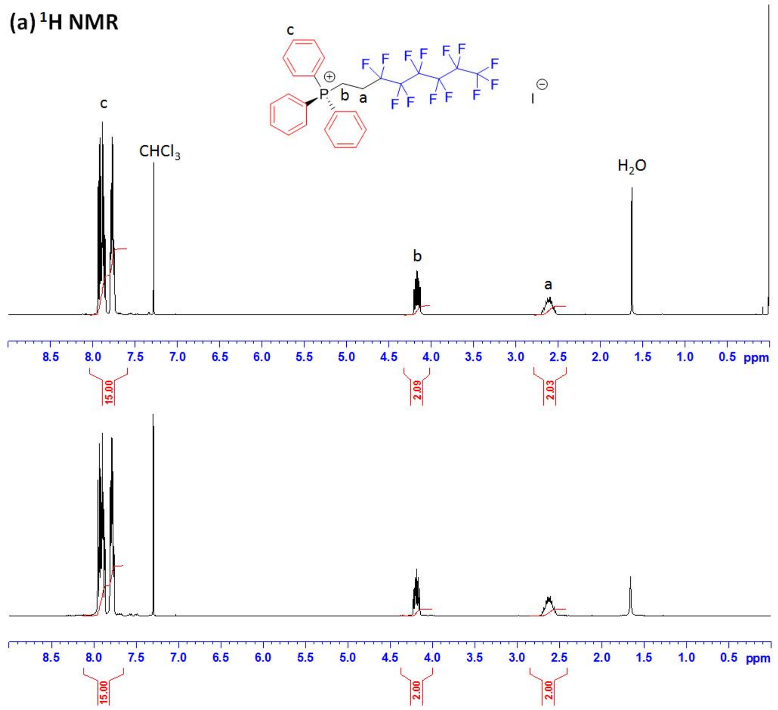

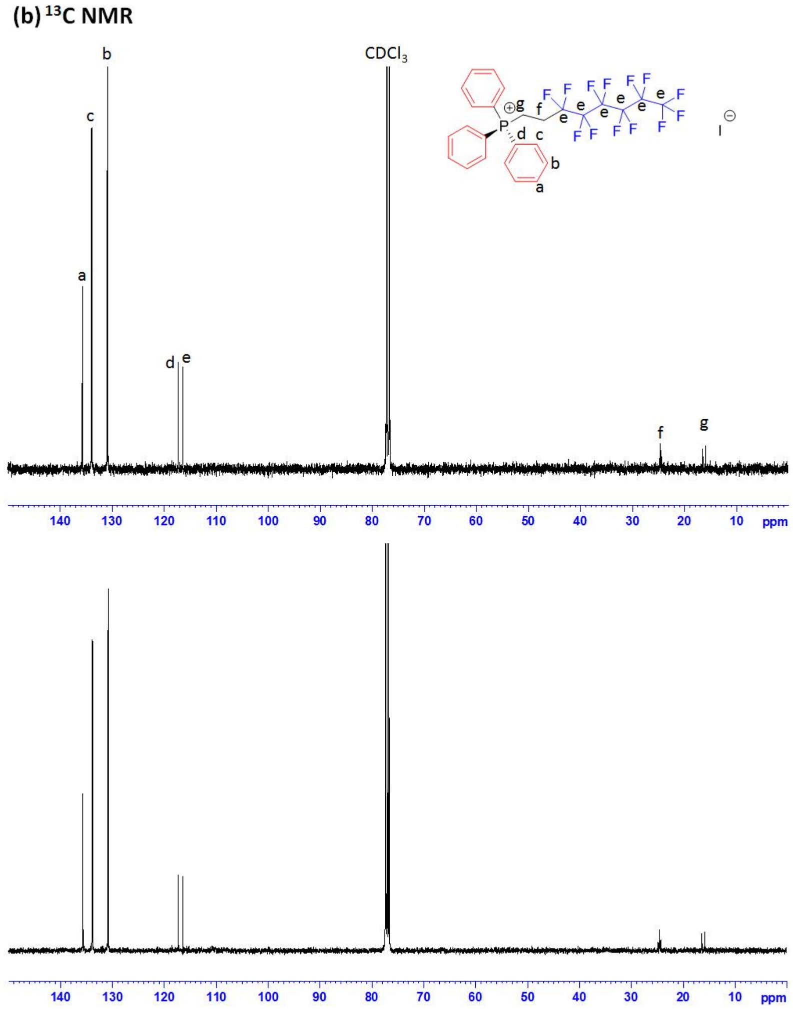

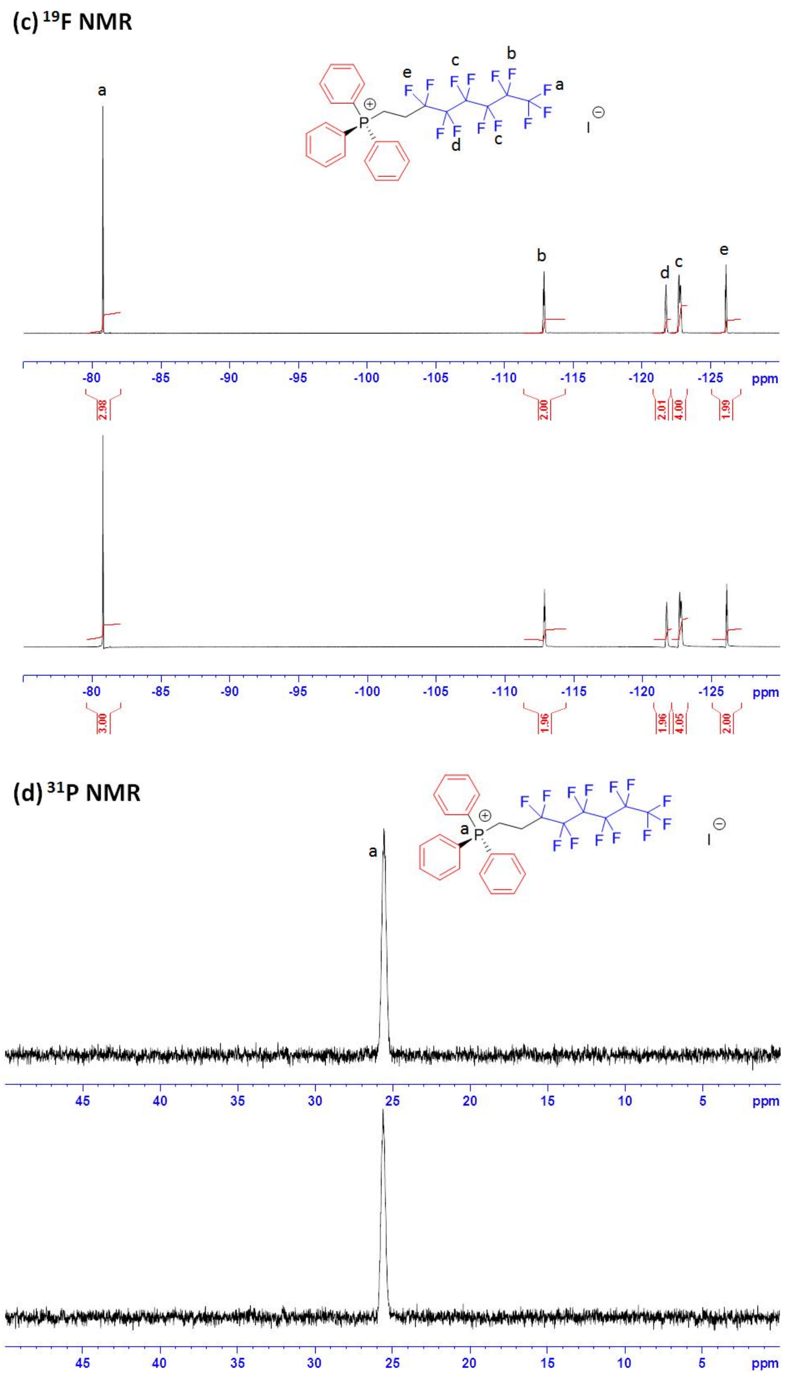

Figure S2. ^{1}H, ^{13}C, ^{19}F and ^{31}P NMR spectra of IL-C8F13 before (upper) and after (lower) hydrazine treatment.

3. DSC traces of neat P(VDF-CTFE) and its composites containing IL, GO, rGO, GO-IL, rGO-IL with different loading contents during heat/cool/heat runs

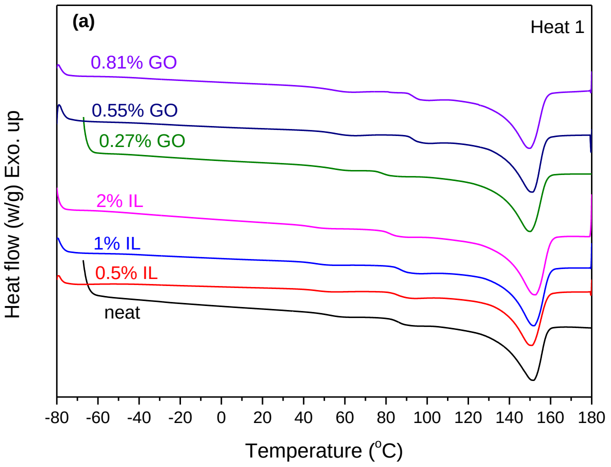

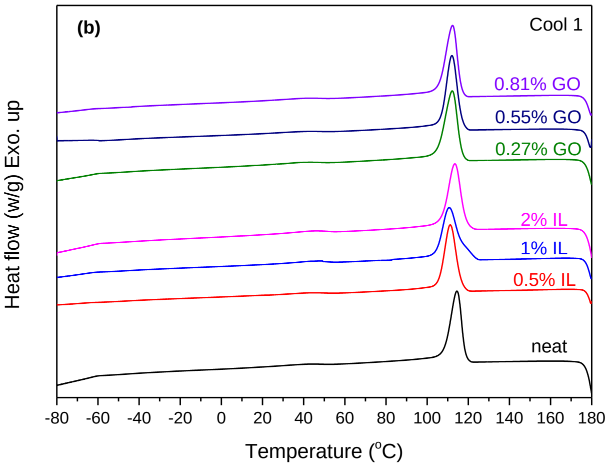

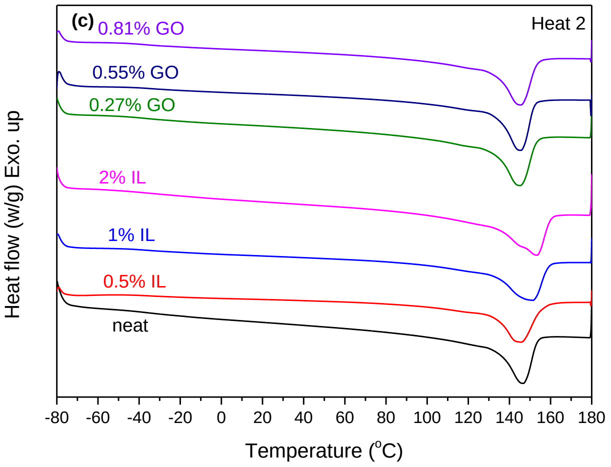

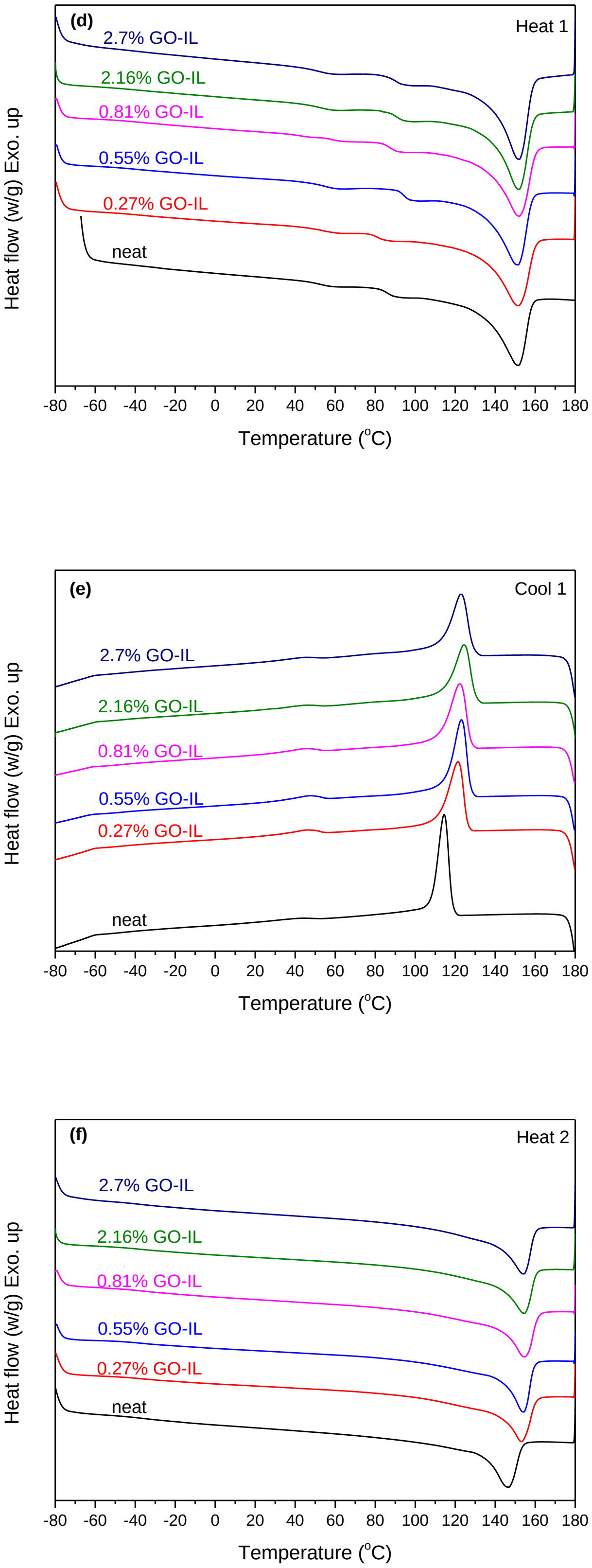
(d)
Heat 1
2.7% GO-IL
2.16% GO-IL
0.81% GO-IL
0.55% GO-IL
0.27% GO-IL
neat
Heat flow (w/g) Exo. up
-80 -60 -40 -20 0 20 40 60 80 100 120 140 160 180
Temperature (°C)
(e)
Cool 1
2.7% GO-IL
2.16% GO-IL
0.81% GO-IL
0.55% GO-IL
0.27% GO-IL
neat
Heat flow (w/g) Exo. up
-80 -60 -40 -20 0 20 40 60 80 100 120 140 160 180
Temperature (°C)
(f)
Heat 2
2.7% GO-IL
2.16% GO-IL
0.81% GO-IL
0.55% GO-IL
0.27% GO-IL
neat
Heat flow (w/g) Exo. up
-80 -60 -40 -20 0 20 40 60 80 100 120 140 160 180
Temperature (°C)

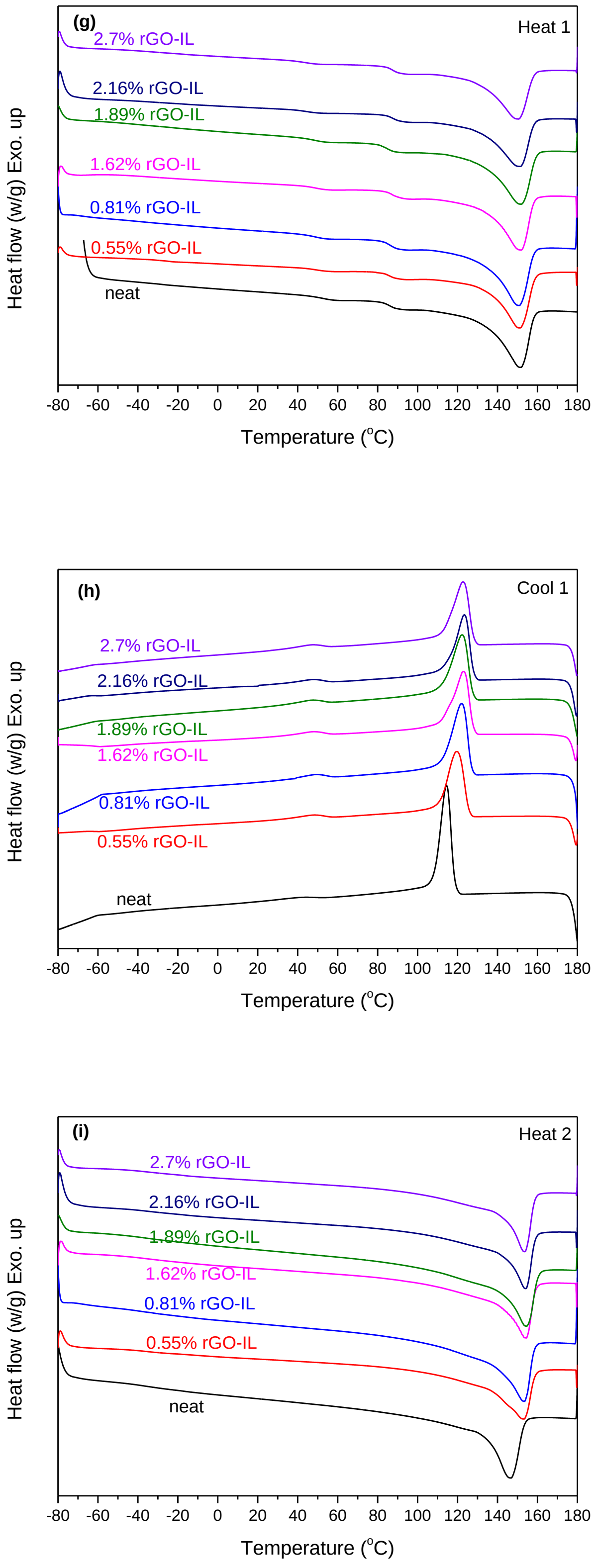

Figure S3. DSC thermograms of neat P(VDF-CTFE) and its composites containing GO, GO-IL and rGO-IL during first and second heating run and the first cooling run.

Table S1. T_c, T_m and ΔH of neat P(VDF-CTFE) and composites from DSC thermograms.

Sample	Heating 1		Cooling 1	Heating 2	
	T_m (°C)	ΔH (J/g)	T_c (°C)	T_m (°C)	ΔH (J/g)
neat	152	32	114	147	30
0.5 wt% IL-C8F13	151	31	111	146	35
1 wt% IL-C8F13	152	34	111	151	35
2 wt% IL-C8F13	153	37	114	153	33
0.27 wt% GO	150	37	112	145	34
0.55 wt% GO	151	31	112	146	35
0.81 wt% GO	150	33	112	146	34
0.27 wt% GO-IL	152	37	121	153	35
0.55 wt% GO-IL	152	34	123	154	35
0.81 wt% GO-IL	152	37	122	155	34
2.16 wt% GO-IL	152	37	124	155	32
2.70 wt% GO-IL	152	40	123	154	34
0.55 wt% rGO-IL	151	33	120	153	32
0.81 wt% rGO-IL	151	35	122	153	32
1.62 wt% rGO-IL	152	32	123	155	30
1.89 wt% rGO-IL	152	35	122	155	30
2.16 wt% rGO-IL	152	30	123	154	30
2.70 wt% rGO-IL	151	31	123	154	30

4. DMA curves of neat P(VDF-CTFE) and its composites containing IL, GO, GO-IL and rGO-IL

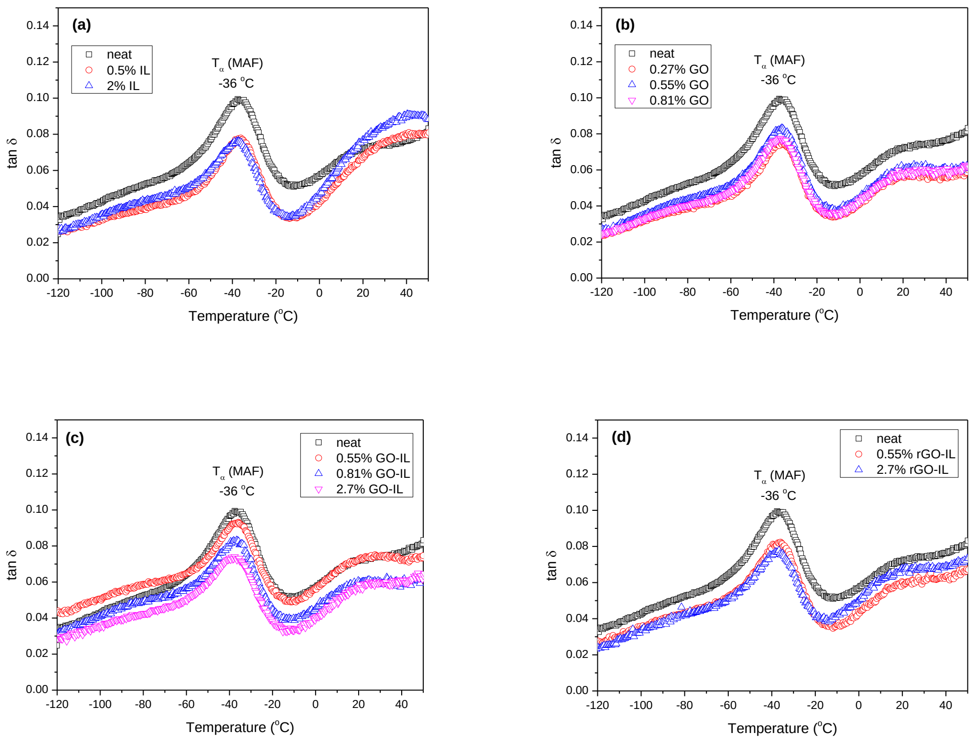

Figure S4. Dynamic mechanical loss tangent (tan δ) as a function oftemperature for neat P(VDF-CTFE) and composites containing IL (a), GO (b), GO-IL (c) and rGO-IL (d).

5. Dielectric properties of P(VDF-CTFE)/GO-IL composites

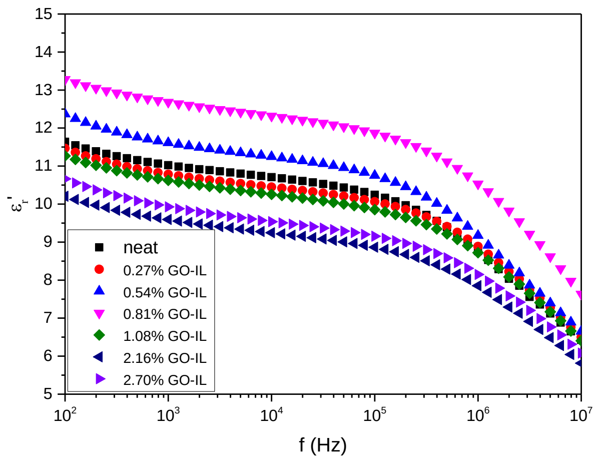

(a)

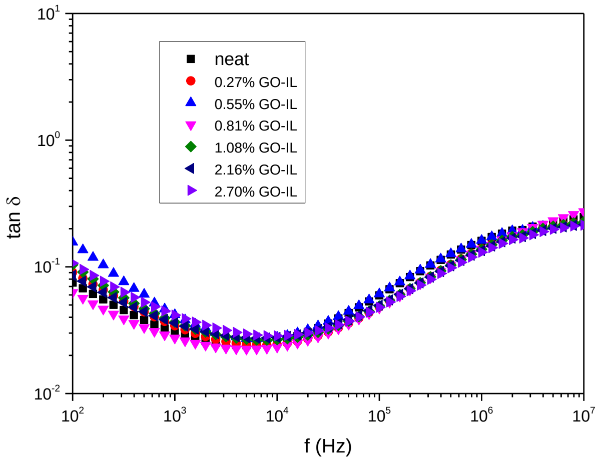

(b)

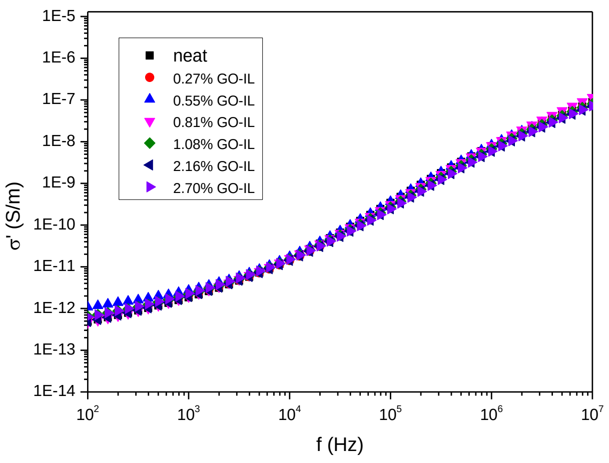

(c)

Figure S5. Relative dielectric permittivity (ε_r') (a), loss tangent (tan δ) (b) and AC conductivity (σ′) (c) of P(VDF-CTFE)/GO-IL composites as a function of frequency at RT with different compositions.
